# Phylogenomic synteny reveals paleohexaploid-derived genomic blocks across Asteraceae

**DOI:** 10.1073/pnas.2426851123

**Published:** 2026-02-10

**Authors:** Tao Feng, Michael McKibben, John Lovell, Richard Michelmore, Loren H. Rieseberg, Michael S. Barker, M. Eric Schranz

**Affiliations:** ^a^Biosystematics Group, Department of Plant Sciences, Wageningen University and Research, Wageningen 6708 PB, The Netherlands; ^b^Department of Ecology and Evolutionary Biology, University of Arizona, Tucson, AZ 85721; ^c^Evolutionary Analysis Group, Genome Sequencing Center, HudsonAlpha Institute for Biotechnology, Huntsville, AL 35806; ^d^Department of Energy Joint Genome Institute, Berkeley, CA 94720; ^e^Department of Plant Sciences and The Genome Center, University of California, Davis, CA 95616; ^f^Department of Botany and Biodiversity Research Centre, University of British Columbia, Vancouver, BC V6T 1Z4, Canada

**Keywords:** paleohexaploid, Asteraceae, genome rearrangement, synteny, ancestral linkage group

## Abstract

The Asteraceae (Compositae) is the largest flowering plant family, ubiquitous in most terrestrial communities, and morphologically diverse. A two-step, ancient whole genome triplication (paleohexaploidization) occurred at approximately the same time as the evolutionary innovation and adaptive radiation of the family during the middle Eocene. Despite its importance, the consequences of this triplication have yet to be tracked in context of the Asteraceae genome evolution. To do so, we applied a synteny oriented phylogenomic analysis of 23 Asterales genomes. We identified 16 genomic groups that date back to the common diploid ancestor of all Asteraceae. Each group underwent triplication, resulting in 48 genomic blocks (16 × 3) that collectively represent the ancestral Asteraceae genome, excluding the early-diverging lineages which do not share the second step. We then analyzed the evolutionary dynamics of the 48 genomic blocks across the Asteraceae phylogeny. We found that modern Asteraceae genomes are genetic mosaics of three progenitor genomes, shaped by genomic exchanges, chromosomal rearrangements, and gene fractionation. One hundred fifty-seven genes retained three paleohexaploid-derived syntenic paralogs across most Asteraceae species. Transcription factors and auxin-related genes are significantly overrepresented in these triplets, and expression of the paleohexaploidy paralogs is spatiotemporally differentiated. These genes are involved in the development of floral capitula, a remarkable morphological innovation of the family. The discovery of the 157 triplicated genes can direct further study to understand the evolutionary innovation, and the synteny-phylogenomic framework provides a comparative framework to characterize newly sequenced Asteraceae genomes.

## Significance

Comparative genomics is a powerful approach for studying the evolution of organisms and their traits. However, deep evolutionary comparisons in plants remain challenging due to the highly dynamic nature of plant genome evolution. In this study, we investigated genome evolution in Asteraceae, the largest family of flowering plants, by reconstructing 16 linkage groups of the paleohexaploid ancestor of all Asteraceae species, dating to ~80 Mya. Our analysis revealed that most modern Asteraceae genomes are mosaics of three progenitor genomes and have undergone extensive reshaping through genome rearrangements and gene fractionation. This phylogenomic synteny framework provides valuable insights into the complex evolutionary history of Asteraceae and provides a window into exploring genome evolution at deep timescales.

The Asteraceae, traditionally known as Compositae, is composed of more than 34,000 species corresponding to ~10% of all flowering plants (https://wfoplantlist.org/; accessed on 12/12/2024). A shared derived feature of all Asteraceae species is the capitulum, a head-like inflorescence mimicking a single flower, which is among the most remarkable morphological innovations in angiosperms after the origin of flowers (1). The Asteraceae are exceptionally diverse in phenotypic traits, specialized metabolites, and ecological habitats, making this family an excellent system to address a broad range of ecoevolutionary questions (2). Asteraceae have colonized almost every conceivable habitat including the harshest ones, for example *Saussurea gnaphalodes* (Royle ex DC.) Sch.Bip. is endemic to the Himalayan periglacial region up to 6,400 m, the uppermost elevation limit recorded for vascular plants (3). There are also species such as the common sunflower (*Helianthus annuus* L.) and dandelion (*Taraxacum officinale* F.H.Wigg.) that are widely distributed. The family is well known for its agriculturally important species such as cultivated sunflower, safflower, lettuce, and globe artichoke and iconic horticultural species such as daisies, gerberas, and chrysanthemums. The hyperdiversity of Asteraceae is suggested to be generated by multiple radiation events during the middle Eocene (4). In addition to their vast morphological and habitat diversity, Asteraceae harbor remarkable genomic variation, with ploidy levels ranging from 1× to 22× (2n = 4 to 198) and 2C-values varying by 139-fold, making it genomically more diverse than many other angiosperm families (5).

Despite the high diversity and species richness of Asteraceae, genome sequencing of the family has lagged, with most sequences being generated only in the past 3 y. Sequencing and assembly of Asteraceae genomes have been challenging because they are generally large due to extensive long and highly similar repeat content (6). However, technical advances in long-read sequencing and bioinformatics have overcome many of these obstacles, bringing about a new era of comparative genomics for the family. The first complete genome sequence of the Asteraceae was for the globe artichoke (*Cynara cardunculus* var. *scolymus* (L.) Fiori) (7), followed by the sequencing of sunflower (*H. annuus*) (6) and lettuce (*Lactuca sativa* L.) (8). As of the time of our analysis, a total of 61 assemblies from 35 distinct species were publicly available (https://www.ncbi.nlm.nih.gov/datasets/genomes/?taxon=4210). There will certainly be an exponential growth of genome sequencing of Asteraceae species in the coming years, highlighting the need for a unified and consistent comparative genomics framework for the family.

Taxonomic and phylogenetic studies of Asteraceae have been challenging, both due to the extremely high species richness but also due to widely occurring hybridization, polyploidization, and rapid radiations (9, 10). However, recent advances in phylogenomic approaches and high-throughput sequencing (including genome, transcriptome, and targeted capture sequencing) have clarified many phylogenetic relationships within the family (4, 10–13). Recent large-scale phylogenomic analyses have established a near-consensus phylogenetic backbone for the family (4, 13) (*SI Appendix*, Fig. S1), with only minor inconsistencies in the placement of several small tribes, likely due to gene tree conflict. Aligning with this backbone, the phylogenetic classification of Asteraceae was recently updated (14), which includes 16 subfamilies and 47 tribes (or 51 tribes if several evolutionary clades are treated as individual tribes). A recent phylogenomic study found Barnadesioideae, a subfamily of Asteraceae, is closer to Calyceraceae (15), making Asteraceae a potentially polyphyletic group.

In addition, phylogenomic studies and genome sequencing have demonstrated widely occurring whole genome duplications (WGD) across the phylogeny, including ancient events during early Asteraceae evolution (12, 13, 16, 17) and more recent events in specific lineages (6, 12, 13, 18, 19). According to these studies, Asteraceae has undergone two successive ancient polyploidization events, the first one shared by Asteraceae and Calyceraceae and the second one shared by all Asteraceae species except for the two first-diverging subfamilies (17, 20). In genomic content, Calyceraceae, Barnadesioideae and Famatinanthoideae are paleotetraploids, while all other extant Asteraceae are paleohexaploids. This two-step hexaploidization was suggested to be a key event in Asteraceae evolution, which might be the main driver of the rise of the group during the middle Eocene (16, 17). However, the contribution of this hexaploidization to the architecture and content of modern Asteraceae genomes is poorly understood. Nonetheless, a robust phylogenetic backbone and the scenarios of genome duplication provide a useful framework for comparative genomic study.

In this study, we aimed to illustrate and understand Asteraceae genome evolution in the context of the paleohexaploidization. We established the first synteny constrained phylogenomic framework for the family using 23 high-quality genomes, from diploid (2n = 2x) to paleotetraploid (2n = 24x) species and included *Scaevola taccada* (Gaertn.) Roxb. (Goodeniaceae) as the nontriplicated outgroup. Using this framework, we aimed to answer 1) to what extent can we trace the genome changes across ~80 My of Asteraceae evolution? and 2) what is the genomic architecture of modern Asteraceae genomes compared with the inferred nontriplicated ancestral genome? Answers to these questions provided insights into Asteraceae genome evolution and its rise to ecological prevalence.

## Results

### Macro- and Microsynteny across Asteraceae Genomes.

To build a synteny phylogenomic framework for Asteraceae, we gathered 30 chromosome-level genome assemblies (*SI Appendix*, Fig. S1 and Table S1). We also included one outgroup genome from the sister family Goodeniaceae. We assessed the quality of the genome data using BUSCO (21) and OMArk (22). OMArk gave slightly different but consistent assessments of the genome assemblies (*SI Appendix*, Table S1). Based on our assessment and considering phylogenetic representation, we selected 23 genome assemblies (OMArk: 90.36 to 98.12; BUSCO: 90.4 to 98.2) for comparative analyses. These assemblies represent 10 tribes across three Asteraceae subfamilies, as well as the outgroup Goodeniaceae.

**Fig. 1.**
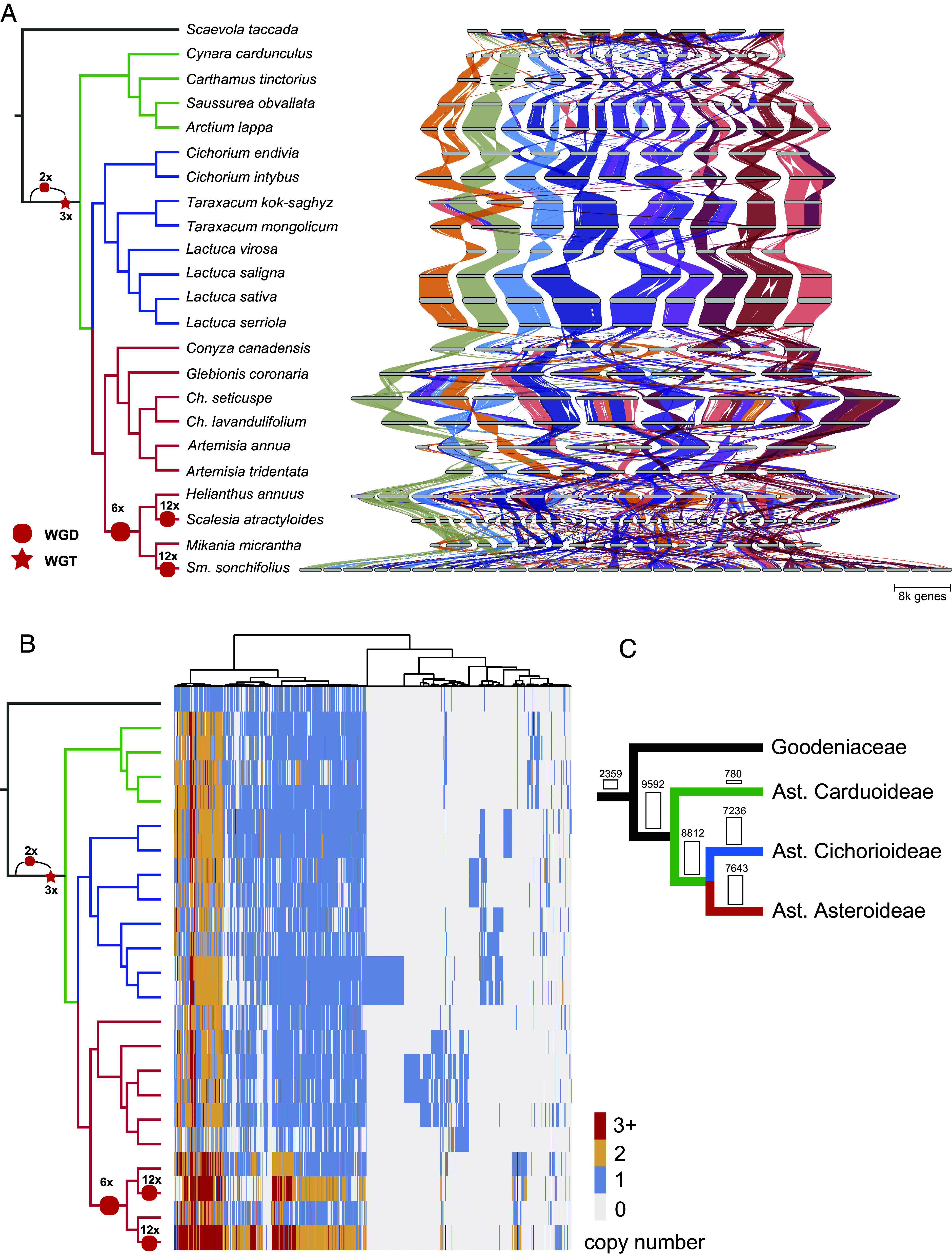
Macro- and Microsynteny across Asteraceae and Goodeniaceae genomes. (*A*) Macrosynteny map (riparian plot) showing orthologous genomic regions across 23 genomes, with the phylogeny adapted from Zhang et al. (13) and branch colors indicating subfamily affiliation: green for Carduoideae, blue for Cichorioideae, and red for Asteroideae. “*Ch*” and “*Sm*” refer to *Chrysanthemum* and *Smallanthus*, respectively. The “2x-3x” notation on the branch marks the two-step wholegenome triplication event shared by most Asteraceae species, including all species used in this analysis. Chromosomes are ordered horizontally to maximize synteny with *Scaevola taccada* and ribbons are color coded by synteny to *S. taccada* chromosomes. (*B*) Phylogenomic synteny profiling of microsynteny gene clusters (size ≥ 2) across 23 genomes. Rows are species in the same order as in *A*, and columns are synteny clusters, each comprising homologous genes that are syntenic across two or more species. Gene copy number is indicated by color bars. Presence/absence patterns are clustered based on Euclidean distance, as indicated by the tree on top; (*C*) Quantitative characteristics of microsynteny clusters shared across Goodeniaceae and three Asteraceae subfamilies that are involved in this study. The numbers on the branches are the counts of synteny cluster shared by the relevant lineages.

With this dataset, we built the first genome synteny map across the Asteraceae and its sister family Goodeniaceae (Fig. 1*A*). Despite the divergence, there was extensive genome synteny across species (Fig. 1*A*). The overall synteny map captured the ancient polyploid events, as seen by the ratios of syntenic regions across the phylogeny (Fig. 1*A*). This includes the ancient polyploidization shared by all Asteraceae species, the ancient duplication shared by *H. annuus*, *Scalesia atractyloides* Arn., *Mikania micrantha* Kunth and *Smallanthus sonchifolius* (Poepp.) H.Rob., and the recent lineage-specific duplications in *S. atractyloides* and *S. sonchifolius* (Fig. 1*A*). Our results support the previous observations based on sequence divergence (*Ks* frequency) and gene tree sorting (12, 17).

Based on this macrosynteny framework we then examined the microsynteny, namely the synteny of individual genes across Asteraceae. We identified 35,832 synteny clusters (*block_size ≥ 5*, *gap_size ≥ 5*, *cluster_size ≥ 2*), of which 2,359 and 9,592 are highly conserved across Asteraceae and Goodeniaceae, and within Asteraceae, respectively (Fig. 1 *B* and *C*). These clusters consist of related genes that remain syntenic across the phylogeny, despite more than 80 My of genome reshuffling, gene fractionation, and transposition. We also detected 7,236, 7,643, and 780 lineage-specific synteny clusters in the subfamilies Asteroideae, Cichorioideae, and Carduoideae, respectively (Fig. 1*C*). Our results also highlight Asteroideae species with higher gene copy numbers per cluster (e.g., orange/red rows in Fig. 1*B*), consistent with the additional WGD events observed in these species (Fig. 1*A*).

### Genomic Architecture of Asteraceae Genomes in Context of Paleohexaploidization.

One of our main objectives is to understand the consequence of the ancient, two-step hexaploidization (ambiguously termed as triplication elsewhere, e.g., refs. 6, 8, and 23) on genome evolution in Asteraceae. To characterize the genome architecture of extant species in context of the paleohexaploidization, we cataloged genomes into genomic blocks. Using *Scaevola taccada* (Goodeniaceae) as an outgroup, we focused on diploid Asteraceae species and quantified genome reshuffling events from *S. taccada* to these species. We used *S. taccada* as the outgroup because it is a close sister lineage to Asteraceae, but it did not experience the paleohexaploidization event and has undergone no additional genome duplications since its divergence (23). On average, 152 fissions and 165 fusions were identified in pairwise comparisons (*SI Appendix*, Fig. S2). The *Arctium lappa* genome is the least rearranged among the genomes analyzed, with 75 inferred fissions and 81 inferred fusions (*SI Appendix*, Fig. S2). The lacking rearrangements in *A. lappa* was further investigated by the comparison of *A. lappa* genome with lettuce genome, in which misassemblies can be ruled out in both genomes (*SI Appendix*, Fig. S3). In addition, the *A. lappa* genome assembly is of high quality in both completeness (BUSCO = 98, OMArk = 97.76) and continuity [LAI = 21.57, gold quality according to the classification based on LTR Assembly Index (24)]. Therefore, *A. lappa* was used as an ingroup reference to compare with the outgroup *S. taccada* to define Asteraceae genomic blocks.

**Fig. 2.**
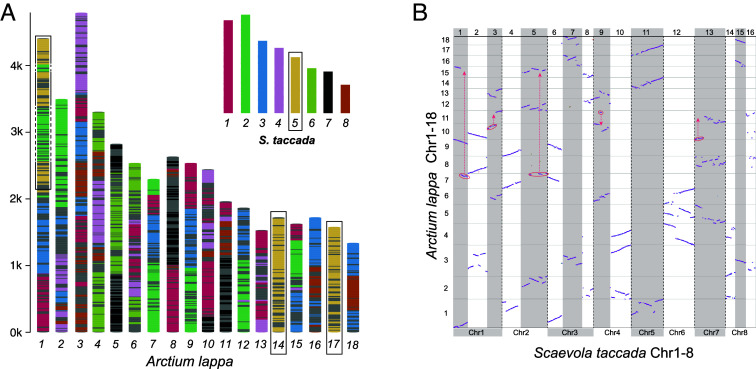
Genomic comparison between *Scaevola taccada* and *Arctium lappa* and the 16 sets of Asteraceae genomic blocks (AGBs). (*A*) The mosaic architecture of *A. lappa* genome with genomic segments colored to show homology to *S. taccada* chromosomes. As an example, *S. taccada* chromosome 5 is homology to *A. lappa* chromosome 14, 17 and top parts of chromosome 1. The chromosome length indicates number of genes rather than nucleotide base pairs; (*B*) Dot plot between *S. taccada* (*x*-axis) and *A. lappa* (*y*-axis) genome. The eight chromosomes of *S. taccada* are divided into 16 regions each aligning with three regions on *A. lappa* chromosomes. The inferred genomic fission/fusion events across different chromosomes of *A. lappa* are highlighted in circles and arrows.

Using the A-Bruijn graph-based algorithm (25), we screened conserved syntenic segments between *A. lappa* and *S. taccada*. In total, 1,324 conserved syntenic segments (at least five genes) were identified in *A. lappa*, of which 291, 460, and 573 segments are in triple, double, and single states (*SI Appendix*, Fig. S4). The average size of the segments is 15 genes, and the largest one has 97 genes. Mapping the 1,324 syntenic segments onto the 18 *A. lappa* chromosomes indicated that the *A. lappa* genome is a mosaic of three progenitor genomes (Fig. 2*A*), as exemplified by chromosome 14 and 17 (homoeologous to chromosome 5 of *S. taccada*), and their paralogous segments are widely distributed across chromosome 1 (Fig. 2*A* and *SI Appendix*, Fig. S4).

Pairwise syntenic comparisons between *S. taccada* and *A. lappa* revealed a clear 1:3 syntenic depth ratio (Fig. 2*B*). Notably, considerable portions of the *A. lappa* genome—particularly chromosomes 14 and 17—have retained largely intact, with only a few inversions relative to the *S. taccada* genome (Fig. 2*B*). This enables us to identify the homoeologous genomic regions that are presumably derived from the ancient genome polyploidization. Following the principles of proximity and complementarity, we assigned the identified genomic blocks of *A. lappa* to 16 sets of homoeologous blocks (blocks with common shared ancestry) (Fig. 2*B* and *SI Appendix*, Fig. S5). Each of the block set contains three blocks that are inferred to be the descendants of the paleohexaploid ancestor and termed as Asteraceae Genome Blocks (AGBs). For example, the chromosome 14, 17, and several segments on chromosome 1 constitute the AGB 11 which is homoeologous to the chromosome 5 of *S. taccada* (Fig. 2*B*).

### Divergence of Asteraceae Genome Blocks (AGBs).

**Fig. 3.**
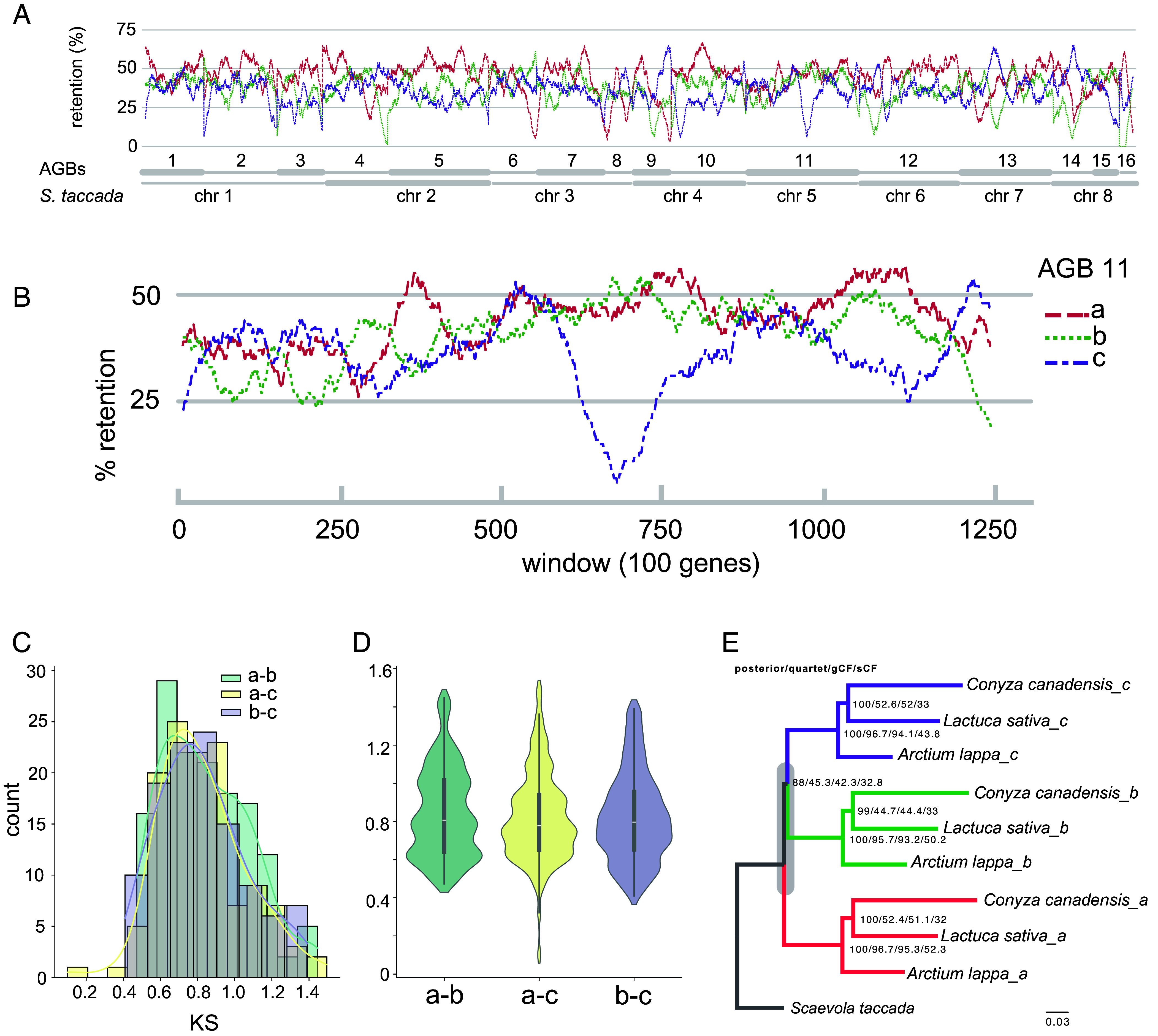
Characterization of AGBs. (*A*) The retention rate of syntenic genes in the three homoeologous blocks of each AGB; (*B*) A zoom-in window of gene fractionation within AGB11; (*C* and *D*) Distribution of synonymous substitution rates (*Ks*) of syntenic gene pairs within AGB11. The *Ks* plots for other AGBs are in *SI Appendix*, Fig. S6; (*E*) Coalescent phylogeny of subgenomes represented by AGB11, based on maximum likelihood (ML) trees of syntenic genes within AGB11. Numbers at nodes show the local posterior probability, quartet support, gene concordance factor (gCF), and site concordance factor (sCF). Stem branch length indicated as ASTRAL coalescent units. The position of genome triplication on the phylogeny is labeled in gray bar.

Next, we used the 16 sets of homoeologous blocks as units to explore the divergence of the subgenomes. To investigate whether there is gene fractionation bias among the three subgenomes, we calculated the gene retention rate in the homoeologous blocks in each set of AGB using *S. taccada* as a reference. Overall, there is no consistent gene fractionation bias across the AGBs (Fig. 3*A* and Dataset S1). As exemplified by AGB11 (Fig. 3*B*), the three homoeologous blocks have ~25 to 55% gene retention rate, and no one is clearly and consistently less fractionated. However, inside the AGBs, for example, the window 1,000 to 1,200 in AGB11, the three blocks are clearly differentiated in terms of gene fractionation, with block *a* being least fractionated and block *c* most fractionated. Window 700 on block *c* is likely a centromere region with low density of protein coding genes (Fig. 3*B*). In addition, we calculated the synonymous substitution rates (*Ks*) using syntenic genes and compared the *Ks* distributions among the homoeologous blocks. There is also no significant divergence in *Ks* distributions among the blocks (*SI Appendix*, Fig. S6), as exemplified by AGB11 (Fig. 3 *C* and *D*).

Initial phylogenetic analysis on the AGBs using a sliding-window approach revealed extensive conflicts among the homoeologous blocks and the outgroup *S. taccada*. For example, on AGB11, 34.7% windows support the topology (*o* (*a*(*b,c*))), while 18.4% and 46.9% windows support alternative topologies (*o*(*b*(*a,c*))) and (*o* (*c*(*b,a*))), respectively (*SI Appendix*, Fig. S7). Through additional phylogenomic analyses with additional species, we confirmed the incongruences in the phylogeny of the three subgenomes. As exemplified by AGB11 (Fig. 3*E*), local posterior probabilities from ASTRAL are consistently high (88 to 100) across all nodes, while concordance factors offer additional insights into phylogenetic incongruence. Notably, the clustering of the three subgenomes across Asteraceae subfamilies is strongly supported by all concordance metrics (with moderate site concordance factors, which is a common pattern also observed in other studies where gene concordance factors and quartet scores are high). This strongly supports the hypothesis that the ancestral genome triplication occurred prior to the divergence of the three subfamilies. In contrast, the clustering of subgenomes *b* and *c* is supported by only 42.3% of genes and 32.8% of sites, indicating that 57.7% of genes and 67.2% of sites support alternative topologies [either (*a*,*c*)*b* or (*a*,*b*)*c*]. Although there are variations in the concordance values among regions, the pattern described above is consistently observed across multiple AGBs (*SI Appendix*, Fig. S8). The homogeneity of the homoeologous genomic blocks and the phylogenetic incongruences hindered further subgenome phasing. Instead of reconstructing biological subgenomes, we assigned the AGBs to three groups (*a*, *b*, *c*) randomly, to provide a comparative genomic framework for characterizing Asteraceae genomes.

### A Synteny-Phylogenomic Framework for Asteraceae.

**Fig. 4.**
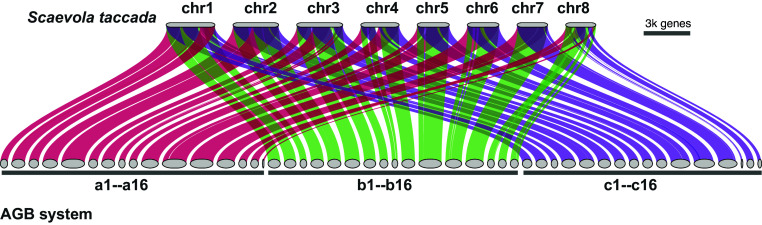
Asteraceae genomic blocks (AGBs). Macrosynteny between the *S. taccada* genome and the AGBs. *a*, *b,* and *c* represent three sets of homoeologous genomic blocks derived from the two-step genome polyploidization in Asteraceae.

Based on the *A. lappa* genome sequence, we reconstructed a model ancestral genome composed of 16 × 3 = 48 AGBs, (Fig. 4 and *SI Appendix*, Fig. S9). This model genome represents the likely genomic architecture and content of the paleohexaploid common ancestor of Asteraceae species. It spans 1.52 Gbp and contains 14,647, 12,205, and 13,135 genes on the three sets of AGBs, respectively (*SI Appendix*, Fig. S9). It is important to clarify that the model genome does not directly represent the ancestral genome of Asteraceae, as *S. taccada* has likely undergone lineage-specific rearrangements since its divergence from Asteraceae. However, because *S. taccada* has not experienced any genome duplication events postdivergence and appears to have undergone fewer genome rearrangements than extant Asteraceae species, we propose that the model genome offers a reasonable approximation of the Asteraceae ancestral genome structure.

**Fig. 5.**
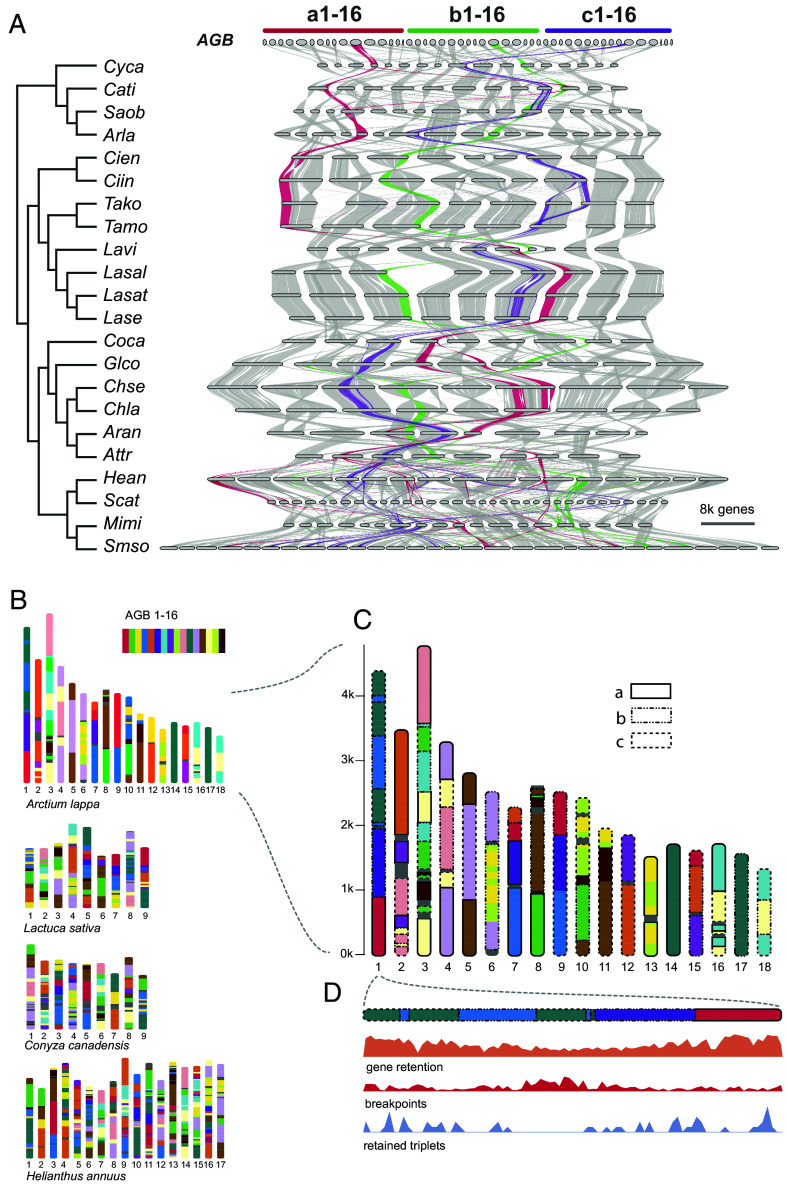
Synteny-phylogenomic framework for Asteraceae. (*A*) Macrosynteny synteny (riparian plot) across 22 Asteraceae genomes and the model genome, with AGB11-a, b, c highlighted in red, green, and purple color to show the dynamics of AGB evolution in Asteraceae. The phylogeny on the left is the same with the one in Fig. 1*A*; (*B*) The genome architecture of four representative species with genomic segments in different colors for tracking the inheritance of AGBs; (*C*) Zooming in on the *Arctium lappa* genome to show the three paralogous regions derived from the same AGB, which labeled in three types of rectangles; (*D*) Zooming in on the chromosome 1 of *A. lappa* with the different genomic features (gene retention, chromosome breakpoints, and RTGs) represented in density along chromosomes by 50-gene window.

Next, we cataloged the Asteraceae genomes in context of the 48 AGBs. We used the model genome as a reference and mapped the Asteraceae genomes onto it using the mutigenome synteny inference approach GENESPACE (26). With this, we generated an updated macrosynteny map across Asteraceae with the ancestral triplicated genomic blocks phased. As shown in the macrosynteny map (Fig. 5 and *SI Appendix*, Fig. S10), the 48 AGBs were recovered in all genomes included in this study, although with further rearrangements in these genomes. This supports the hypothesis of the ancient genome triplication that shared by most Asteraceae species. Therefore, most extant Asteraceae genomes, like *A. lappa* genome, are mosaics of three progenitor genomes. Given any genomic region or genes of interest, for example, the AGB11 (highlighted in Fig. 5*A*), the evolutionary dynamics can be traced across Asteraceae phylogeny. This cataloged genomic resource of 22 Asteraceae species and the outgroup *S. taccada*, including macrosynteny (*SI Appendix*, Fig. S10) and microsynteny (*SI Appendix*, Fig. S11), provides a framework to conduct comparative phylogenomic study in Asteraceae. We have developed a pipeline (https://github.com/xiaoyezao/Asteraceae-synteny-phylogenomics) for mapping genomes onto the 48 AGBs. Therefore, newly generated genome sequences can be readily incorporated into this framework.

### One Hundred Fifty-Seven Genes Retain Three Syntenic Paleoparalogs.

To further understand the consequence of polyploidization on gene content, we analyzed gene fractionation, a process by which gene copies are eliminated from homoeologous segments—namely the AGBs—after polyploidization. Overall, there is no clear gene fractionation bias among subgenomes in the level of the AGBs identified here. Taking AGB11 as an example, on average 55%, 57%, and 64% genes (per 100-gene window) have been lost in *a*, *b,* and *c*, respectively (Fig. 3*B* and *SI Appendix*, Fig. S12), and this is consistent across species (*SI Appendix*, Fig. S12). However, inside the AGBs, for example window 1,000 to 1,200 in AGB11, the three blocks are clearly differentiated in terms of gene fractionation, with subgenome *a* being least fractionated and subgenome *c* most fractionated (Fig. 3*B*). Gene retention across the three homoeologous AGB regions exhibits a reciprocal pattern, where higher retention in one subgenome is often accompanied by lower retention in the other two subgenomes within the same genomic window (*SI Appendix*, Fig. S12). This pattern of differential fractionation leads to an average combined retention rate of ~124% per region across the three subgenomes, out of a theoretical maximum of 300% based on triplicated ancestral gene content in Asteraceae.

The retention of 124% paleohexaploidy homologs in modern Asteraceae species indicates that gene fractionation has eliminated most paleoparalogous copies. However, 24% of paleoparalogs have been retained after 80 My of Asteraceae evolution. To independently validate patterns of fractionation, we ran the machine learning tool Frackify (27) on 14 diploid Asteraceae genomes. These analyses recovered that on average 23% of each genome were paleoparalogous genes, and each species contained on average 860 triple-copy paleoparalogs (*SI Appendix*, Table S2), consistent with our synteny-based analyses.

**Fig. 6.**
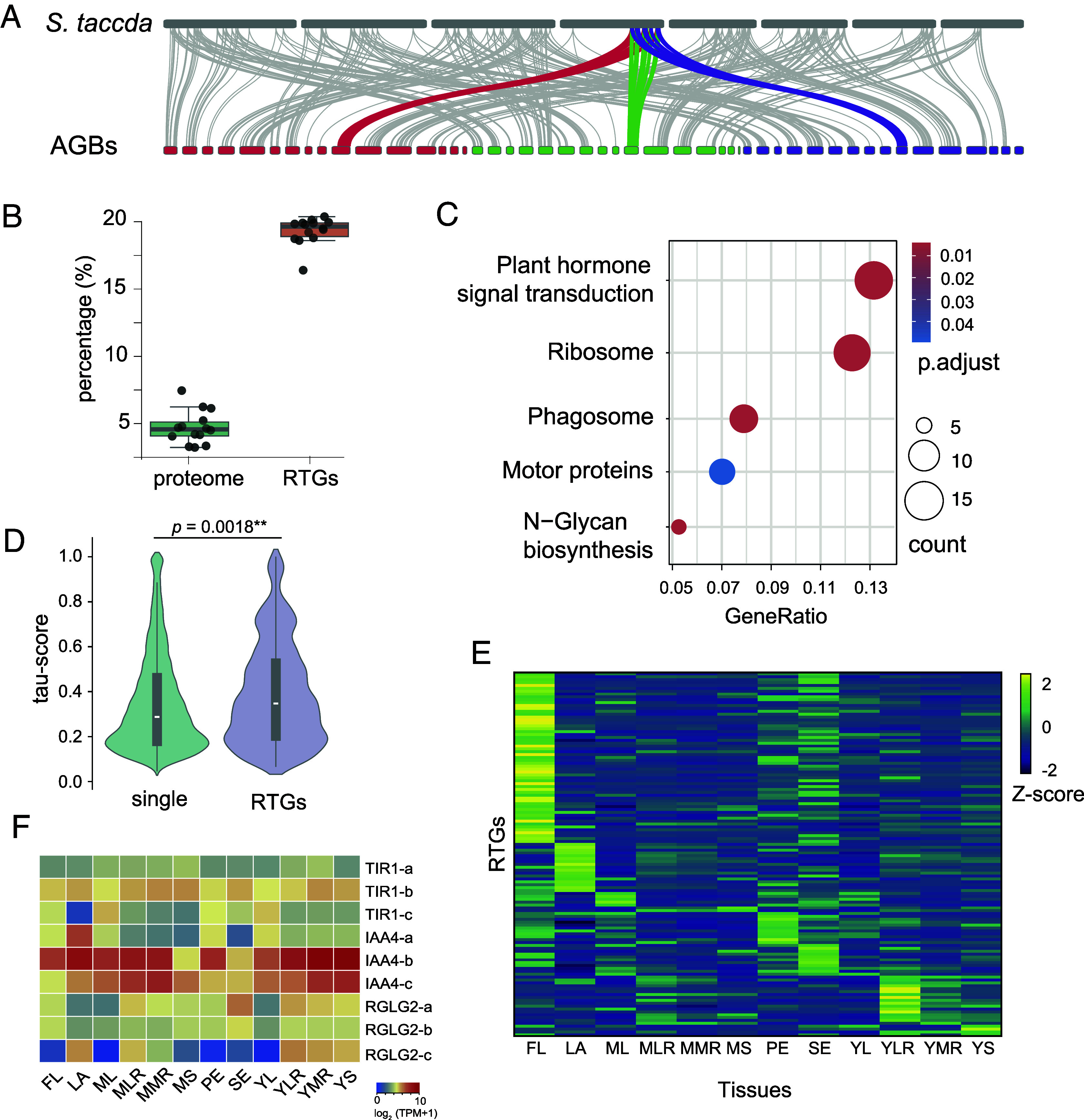
The retained triplicated genes (RTGs). (*A*) Distribution of RTGs on *S. taccada* and the model genome and their syntenic relationships, with a hotspot of RTGs on the end of chromosome 4 of *S. taccada* highlighted; (*B*) The percentage of transcription factors (TFs) in proteomes and RTGs in Asteraceae species; (*C*) Enriched KEGG pathways of the RTGs; (*D*) Expression specificity (measured as *tau* score) of RTGs and single-copy genes in dandelion (*Taraxacum kok-saghyz*); (*E*) The expression of RTGs in 12 different tissues of dandelion (FL-flower, LA-latex, ML-mature leaf, MLR-mature lateral root, MMR-mature main root, MS-mature stem, PE-peduncle, SE-seed, YL-young leaf, YLR-young lateral root, YMR-young mature root, YS-young stem. The expression was measured as TPM value and normalized as z-score; (*F*) Expression of auxin related genes in various dandelion tissue.

The triple-copy paleoparalogs identified in Asteraceae species represent an important genetic legacy of ancient polyploidization and may have contributed substantially to the evolutionary history of the family. To further investigate the paleoparalogs in context of Asteraceae phylogeny, we performed multispecies comparison and focused on the genes that have all three paleoparalogs retained across species. Under different thresholds, the number of the triple-copy genes varies slightly (*SI Appendix*, Fig. S13). However, there are 157 genes (471 paleoparalogs) consistently retained across the three Asteraceae subfamilies studied here, with some copies missing in a few species (Fig. 6*A* and Dataset S2). It is likely that these genes are also retained in other Asteraceae lineages because the sampling here covers diverse evolutionary lineages except for the early-diverging ones (*SI Appendix*, Fig. S1). These genes are termed as retained triplicated genes (RTGs), hereafter. Further investigation of the RTGs indicates that they are distributed across the AGBs (Fig. 6*A*) and form several hotspots, such as on AGB10 (Fig. 6*A* and *SI Appendix*, Fig. S14).

### RTGs Are Overrepresented by Transcription Factors and Enriched in Plant Hormone Pathways.

Next, we ask what types of genes are the RTGs, and what is the evolutionary significance of their conservation? Initial inspection of the RTGs found that many of them are transcription factors (TFs), such as *MYB*, *WRKY,* and *NAC* (*SI Appendix*, Table S2). We then screened the proteomes of the Asteraceae species and identified 1,705-2,650 TFs (https://planttfdb.gao-lab.org/prediction.php, accessed at 26-05-2024), which on average make up 4.1% of the proteomes. However, on average 18.5% of the 471 RTGs are TFs, which is significant overrepresentation (Fig. 6*B*). Gene function enrichment analysis identified four significantly overrepresented KEGG pathways, with the top one being plant hormone signaling pathways, specifically the auxin and ethylene related biological processes (Fig. 6*C*). Further gene ontology (GO) enrichment analysis found that the RTGs are significantly enriched in 179 GO terms (*P* < 0.05) which are clustered into several modules, including flower development and plant hormones (*SI Appendix*, Fig. S15).

We then explored the expression specificity and divergence of the RTGs in various tissues of Russian dandelion (*Taraxacum kok-saghyz* L.E.Rodin) (28). We found that compared to single-copy genes, the RTGs are significantly specialized in expression pattern, namely the expression of RTGs has higher tissue specificity (Fig. 6*D*). Interestingly, almost half of the RTGs (*tau* > 0.5) are preferentially expressed in flowers (Fig. 6*F*). Consistently, there are 30 RTGs of which the *Arabidopsis* orthologs are known to be involved in flower development (Dataset S2), including the MADS domain transcription factor *SOC1* which is a central floral integrator (29). There are also several diverse auxin-signaling related genes which show differentiated gene expression between the triplicated paralogs (Fig. 6*E*). This includes *TIR1* (the auxin receptor whose function is to mark Aux/IAA’s for degradation in the presence of auxin), *IAA4* (a repressor of the auxin response and turned over by *TIR1*), and *RGLG2* (a RING domain ubiquitin E3 ligase targeting auxin pathway proteins for degradation).

## Discussion

The genome triplication of the Asteraceae has been accepted to have occurred via a two-step process (12, 17, 20). However, the contribution of progenitor genomes to modern Asteraceae genomes and the biological significance of this paleohexaploidization require further research. Initial studies of this event relied on distant comparisons between Asteraceae and *Vitis vinifera* (20) or were limited to pairwise comparisons of specific regions (23). Our phylogenetically supported multigenome synteny analysis, allows us to investigate genomic conservation and diversification at multiple scales.

At the chromosome level, we observed that the progenitor genomes had evolved through extensive fissions, fusions, inversions, and translocations, resulting in the modern Asteraceae genomes being complex mosaics, with variability evident between lineages. Such highly dynamic evolution is different from the recently sequenced paleohexaploid *Platanus* genome which has retained almost the same karyotype as its ancestor (30). Chromosomal rearrangements are hypothesized to facilitate adaptation and genomic innovation, both by bringing together previously unlinked adaptive alleles and by creating regions of low recombination that facilitate the linkage of adaptive alleles (31). The high homogeneity among homoeologous blocks and the observed phylogenetic incongruences in subgenome relationships suggest that the three sister AGBs experienced extensive genomic exchange in the paleohexaploid ancestral genome. This likely occurred through recombination between homologous regions during and after the two-step genome triplication. Although incomplete lineage sorting (ILS) cannot be entirely excluded, its impact is probably limited, as the phylogenetic inference was based solely on syntenic orthologs. Given the ancient nature of the genome triplication event in Asteraceae, the resulting genomic exchanges would have been inherited by all descendants of the paleohexaploid ancestor, including the species analyzed in this study. Such exchanges among the progenitor genomes may have further contributed to the remarkable genomic diversity observed across Asteraceae.

At the gene level, we identified Asteraceae-wide and lineage-specific synteny clusters. The evolutionary significance of gene synteny is not well understood; however, evidence for the coregulation of neighbor genes is accumulating (32, 33). Colocalization of genes in genomes may facilitate preservation of favorable allele combinations between epistatic loci or coregulation of functionally related genes (34). One of the most outstanding examples is the biosynthetic gene clusters which have been widely reported in plant specialized metabolisms (32, 35). Additionally, we found there is no significant gene fractionation bias among progenitor genomes in Asteraceae, suggesting that they have contributed equally to the gene content of Asteraceae. However, further comparative genomic analysis involving the intermediate paleotetraploids, such as Calyceraceae, is needed to understand the nature of the two-step triplication. In short, we demonstrated that the two-step genome triplication, together with subsequent genomic exchanges, chromosomal reshufflings, and gene fractionation, collectively reshaped the architecture and gene content of modern Asteraceae genomes.

We also highlighted the RTGs identified in Asteraceae genomes. Different from previous work based on pairwise comparison (23), our comparative genome analysis includes multiple genomes from diverse lineages, therefore accounting for lineage-specific gene loss. We found that the RTGs are significantly overrepresented by TFs, mainly plant hormone-related regulatory genes. A previous study on the retention of Asteraceae paleologs based on evolutionary distance (*K*s) of gene pairs found that transcription and other regulatory functions were significantly underrepresented (16). It is likely that the *K*s-based paleologs include both triplicated and duplicated genes, and nonsyntenic genes, representing an assemblage of more broad paralogs than the RTGs identified here. Additionally, many of the RTG paleologs exhibit organ-specific expression, as exemplified by the Russian dandelion, suggesting subfunctionalization. Our study offers a unique perspective on the conservation and diversification of paleologs in Asteraceae. Together, these observations indicated that retention of the triplication-derived paleologs in Asteraceae is heterogenous among functional categories.

Understanding the genetic basis of morphological innovation in the Asteraceae, such as the capitulum, is a major goal of the Asteraceae community (36, 37). The origin of complex organs can be driven by rewiring of existing regulatory networks (38). For example, the origin of roots in vascular plants has been attributed to the recruitment of IC-WOX genes which perform different functions in nonvascular plants (39). It is suggested that the morphogenesis of the flower-like capitulum in Asteraceae is driven by recruitment of existing conserved developmental regulators (37). Intriguingly, genes annotated as floral development are significantly enriched among the RTGs; for example, *SOC1*, a MADS TF at the center of the flowering network (29), has retained all three paleologs across representative Asteraceae subfamilies. Genes related to plant hormones are also prominent; notably, several auxin-associated genes, which play an essential role in the development of the Asteraceae capitulum (36, 40, 41), have likewise preserved their copies. Given the widespread occurrence of floral heteromorphy in Asteraceae (42–44), it is reasonable to hypothesize that subfunctionalization of the associated RTG paleoparalogs may have played a role in determining floret identity. Notably, half of the RTGs display tissue-specific expression, predominantly in flowers, as illustrated by Russian dandelion. Understanding the conservation or diversification of the RTGs across Asteraceae lineages, can provide insights into the evolution of the regulatory network(s) underlying capitulum morphogenesis, and the phenotypic diversity of capitula.

Given the considerable variability in genome architecture between lineages and the high evolutionary dynamics of AGBs in Asteraceae, phylogenetic coverage is crucial for future comparative genomic analysis. Current Asteraceae genomic data are biased toward certain taxonomic groups and hence are phylogenetically uneven, with only 10 of the 57 tribes represented (and eight of them from only the subfamily Asteroideae). Early-diverging lineages, such as Barnadesioideae and Famatinanthoideae, should be a priority for future sequencing projects. Nonetheless, our synteny-phylogenomic framework, the 48 AGBs, and a suite of tools to interpret whole genome synteny and regional syntenic clusters should accelerate future comparative genomic studies of the Asteraceae.

## Methods

### Genome Data and Quality Assessment.

As of the time of our analysis, 61 assemblies from 35 species were publicly available (https://www.ncbi.nlm.nih.gov/datasets/genomes/?taxon=4210), most of which were generated within the past 3 y. As our main objective was to build a synteny-phylogenomic framework for Asteraceae, we applied several criteria for selecting genomes for comparative analysis: 1) chromosome level assembly; 2) high BUSCO and OMArk scores; and 3) maximized phylogenetic representation. This resulted in the inclusion of 22 Asteraceae genomes from 10 tribes across three subfamilies (*SI Appendix*, Fig. S1 and Table S1). In addition, *Scaevola taccada* (Goodeniaceae) was included as an outgroup. Full details of the BUSCO and OMArk analyses are provided in *SI Appendix*.

### Multigenome Macrosynteny.

Global synteny across the 23 genomes was inferred using GENESPACE v1.3.1 (26). Based on prior knowledge of the genome duplication history and ploidy level of Asteraceae species (*SI Appendix*, Table S1), we carried out three analyses to address different objectives. Analysis 1: construction of genome synteny between the outgroup *S. taccada* and Asteraceae species; Analysis 2: construction of synteny among Asteraceae species; Analysis 3: because we are specifically interested in orthologous synteny between *S. taccada* and Asteraceae species, we used the same hierarchical orthologous groups (hOGs) as in Analysis 1 but set the ploidy level to “1” for all species. This approach recovered the same orthologous synteny as Analysis 1 while excluding paralogous synteny within Asteraceae. Full details of these analyses are provided in *SI Appendix*. Macrosynteny across species was visualized using a riparian plot.

### Multigenome Microsynteny.

Microsynteny refers to the local conservation of gene order across genomes. With the macrosynteny results described above, we obtained syntenic genes using the *query_pangene* function in GENESPACE. Syntenic gene pairs were then generated using a custom script and were used as input for *syntenet* v1.1.6 (45) to identify microsynteny clusters under default settings, and to perform phylogenetic profiling using the function *phylogenomic_profile*. Microsynteny clusters (size ≥ 3) across Asteraceae and Goodeniaceae genomes were visualized as copy-number heatmaps (Fig. 1*B* and *SI Appendix*, Fig. S11) using the *plot_profiles* function with default settings. In addition, we quantified the microsynteny gene clusters along the phylogenetic branches leading to the major clades of Asteraceae.

### Chromosome Rearrangements.

*Scaevola taccada* was used as an outgroup to infer the chromosomal rearrangements in diploid Asteraceae species (*SI Appendix*, Table S1). Comparisons between *S. taccada* and the Asteraceae species allow the inference of the diploidization processes that occurred in Asteraceae genomes after the paleohexaplodization event, providing that *S. taccada* did not experience additional genome duplications following its divergence from Asteraceae. To infer genomic rearrangements (fusions, fissions, inversions, and translocations), syntenic genes identified in the microsynteny analysis were used as anchors to reconstruct synteny blocks with DRIMM-Synteny (25) (*cycleLengthThreshold* = 20, *dustLengthThreshold* = 5), based on a syntenic depth ratio of 1:3. Genomic blocks shared between *S. taccada* and Asteraceae species that contained at least five anchor genes were then used to infer rearrangement events using IAGS (*-can 1*, *-ccn 3*) (46).

### Reconstruction of Asteraceae Genomic Blocks (AGBs).

We used *A. lappa* to reconstruct the AGBs because its genome exhibits the fewest rearrangements in our dataset (*SI Appendix*, Fig. S2) and this lack of rearrangements is likely genuine rather than an artifact of misassembly (*SI Appendix*, Fig. S3). To reconstruct the AGBs, several rounds of adjustment and optimization of genomic blocks were performed. First, the synteny segments recovered from the DRIMM-Synteny analysis were used as level 1 blocks. Second, adjacent level 1 blocks (size ≥ 3) were merged into longer blocks if their counterparts in the *S. taccada* genome were physically adjacent. This approach assumes that these syntenic segments share a common ancestry and belong to the same linkage group. As a result, 98 level 2 blocks were generated (*SI Appendix*, Table S3), covering 90.15% of the *A. lappa* genome. Third, the level 2 blocks were assigned to 16 × 3 blocks (level 3) based on the principles of proximity and complementarity, guided by their alignment to the *S. taccada* genome (*SI Appendix*, Fig. S5). The level 3 blocks are interpreted as representing the three subgenomes derived from the ancient Asteraceae genome polyploidization event. Because these blocks were reconstructed using the extant *A. lappa* genome, lineage-specific rearrangements may have altered the chromosome architecture relative to the ancestral state. Finally, to approximate the ancestral configuration, we used *S. taccada* as a reference and rearranged the level 3 blocks in both orientation and order to maximize collinearity with the *S. taccada* chromosomes (*SI Appendix*, Fig. S9), which give rise to the level 4 blocks.

In addition, we carried out phylogenomic and *Ks* analyses (*SI Appendix*) to assign the level 4 blocks to the three subgenomes. However, precise subgenome phasing was not feasible given the extensive genomic changes among homoeologous chromosomes, as indicated by both the phylogenomic and *Ks* analyses (*SI Appendix*). Instead, we assigned the level 4 blocks into three groups randomly, each containing 16 blocks, and the three groups were designated as the Asteraceae genomics blocks (AGBs).

### Gene Fractionation.

Gene fractionation in the Asteraceae genomes was first analyzed by comparing the AGBs with the *S. taccada* genome. The AGBs were aligned to the *S. taccada* genome as described in the macrosynteny analysis. Syntenic genes were obtained using the *query_pangene* function in GENESPACE, with *S. taccada* as the reference. Gene fractionation within the AGBs was characterized by calculating the retention of syntenic genes in each AGB within sliding 100-gene windows along the reference chromosomes. Fractionation in the Asteraceae genomes (14 of the 22 genomes show no evidence of additional WGDs after the ancient WGT) was calculated following the same procedure.

### Identification of Retained Triplicated Genes (RTGs).

To identify genes that retained all three paleoparalogs derived from the ancient genome triplication—referred to as retained triplicated genes (RGTs)—we performed a multispecies gene fractionation analysis (*SI Appendix*). The analysis allows for the simultaneous examination of whether a given gene retains all three paleoparalogs across multiple species. To focus on the evolutionary consequences of the ancient WGT, the analysis was limited to 14 of the 22 Asteraceae genomes that show no evidence of subsequent WGDs. The results were further verified using the Frackify pipeline (*SI Appendix*).

### RTGs Gene Expression and Gene Ontology.

RNAseq data from Russian dandelion (28), covering diverse tissues and developmental stages, were used to explore the divergence and conservation of the RTGs. We quantified gene expression and expression specificity (*SI Appendix*) of the RTGs to characterize the paleoparalogs. In addition, we annotated the RTGs and examined their biological functions using GO and KEGG enrichment analyses (*SI Appendix*).

## Supplementary Material

Appendix 01 (PDF)

Dataset S01 (XLSX)

Dataset S02 (XLSX)

## Data Availability

Scripts data have been deposited in GitHub (https://github.com/xiaoyezao/Asteraceae-synteny-phylogenomics) (47).
