# Supplementary material for: Phylogenomic synteny reveals paleohexaploid-derived genomic blocks across Asteraceae
Source: Proc Natl Acad Sci U S A. 2026 Feb 10;123(7):e2426851123. doi: 10.1073/pnas.2426851123 (PMC12912976; doi:10.1073/pnas.2426851123)
Supplement: Supplementary file 1 — Appendix 01 (PDF) [file pnas.2426851123.sapp.pdf]

PNAS

Supporting Information for

Phylogenomic synteny reveals paleohexaploid-derived genomic blocks across Asteraceae

Tao Feng[a,1,2], Michael McKibben[b], John Lovell[c,d], Richard Michelmore[e], Loren H. Rieseberg[f], Michael S. Barker[b], and M. Eric Schranz[a,1]

[a]Biosystematics Group, Department of Plant Sciences, Wageningen University, Droevendaalsesteeg 1, 6708PB Wageningen, The Netherlands
[b]Department of Ecology & Evolutionary Biology, University of Arizona, Tucson, AZ 85721, USA
[c]Evolutionary Analysis Group, Genome Sequencing Center, HudsonAlpha Institute for Biotechnology, Huntsville, AL 35806, USA
[d]Department of Energy Joint Genome Institute, Berkeley, CA 94720, USA
[e]Department of Plant Sciences and The Genome Center, University of California, Davis, CA 95616, USA
[f]Department of Botany and Biodiversity Research Centre, University of British Columbia, Vancouver, BC V6T 1Z4, Canada

[1]To whom correspondence should be addressed. Email: tf326@cam.ac.uk or eric.schranz@wur.nl

[2]Present address: Department of Plant Sciences, University of Cambridge, Downing Street, Cambridge CB2 3EA, United Kingdom

This PDF file includes:

Supporting text
SI Tables S1 to S3
SI Figures S1 to S15
Legends for Datasets S1 and S2
SI References

Other supporting materials for this manuscript include the following:

Datasets S1 and S2

Supporting Information Text

BUSCO and OMArk analyses

BUSCO analyses were performed using the eudicot single-copy genes (*eudicots_odb10*) as a reference and conducted in BUSCO v5.8.2 (1) with default settings. Because our analyses largely rely on the quality of the annotated protein-coding regions (proteomes), we further assessed proteomes quality using OMArk v0.3.0 (2), which assigns query protein sequences into known gene families and compares them to the expected families of the species' lineage. Therefore, OMArk evaluates not only completeness but also the overall consistency of the gene repertoire relative to closely related species and identifies likely contamination events. OMArk analyses were carried out with default settings, except that "asterids" was used as the reference lineage, and the precomputed asterids orthologous groups was downloaded from OMA (https://omabrowser.org/oma/home/; accessed 05-26-2023).

GENESPACE analyses

GENESPACE utilizes OrthoFinder to first infer hierarchical ortholog groups (hOGs), based on which the homologous genomic regions between genomes—namely, syntenic blocks—are identified. To infer hOGs, we run OrthoFinder v2.5.5 (3) on the 23 genomes (proteomes) using a predefined species tree. The exact OrthoFinder settings were: *"-M msa -S diamond -A mafft -I 1.5"*. The OrthoFinder results were then used as inputs for GENESPACE v1.3.1 to perform three analyses.

Analysis 1: To construct genome synteny between the outgroup *S. taccada* and Asteraceae species, hOGs at node N1 (Fig. S1) were used. The ploidy level for *S. taccada* was set to '1', while ploidy level for Asteraceae species was set to '3' for diploids and '3x' for extant polyploids or extant diploids that experenced additional WGD events (Table S1), where x represents the ploidy level or number of additional WGD after the ancient triplication. For example, sunflower is an extant diploid but has undergone one WGD after the ancient triplication, so its ploidy level is '6'.

Analysis 2: To construct the synteny among Asteraceae species, hOGs at node N2 were used. Ploidy level for all diploids were set to '1', and '1x' for species with additional WGDs as mentioned above.

Analysis 3: Because we were specifically interested in the orthologous synteny between *S. taccada* and Asteraceae species, we used the same hOGs as in Analysis 1 but set the ploidy level to '1' for all species. This recovered the same orthologous synteny as in Analysis 1 while excluding paralogous synteny among Asteraceae species.

For the synteny analyses, protein sequences shorter than 30 amino acids, and hOGs scattered across ≥ 24x locations (where x is the ploidy level for a given species) were included in OrthoFinder analysis but excluded from synteny inference. We used 24x, rather than the default 8x because the ingroup species underwent genome triplication relative to the diploid outgroup. To infer synteny blocks, both the minimum number of anchor genes required to define a collinear block, and the maximum number of intervening genes allowed between anchors (i.e., gap size) were set to five (*block_size = 5, gap_size = 5*), following the developer's recommendation (4).

Phylogenetic analysis

Phylogenetic analyses were conducted for each set of the 16 AGBs using the syntenic genes. In the initial analyses, we performed sliding-window phylogenetic inference using *S. taccada* and *A. lappa*. Coding sequence windows (size = 5 genes, step = 5 genes) were generated for each AGB, and windows containing at least two genes in any subgenomes and *S. taccada* were used for phylogenetic inference. Concatenated protein sequence alignments for each window were generated using MAFFT v7.520 (*--genafpair; --maxiterate 1,000*) (5), and cleaned with trimAl v1.4.1 (*-gt 0.6; -st 0.001*) (6). The best-fit amino acid substitution model was identified using ModelFinder (7), and the best maximum likelihood tree was inferred using RAxML v2.3.6 (8) with 200 bootstrapping replicates. Topological inconsistences were visualized using DensiTree (9).

We further conducted more comprehensive phylogenomic analyses with expanded sampling, including *Lactuca sativa* (subfamily Cichorioideae) and *Conyza canadensis* (subfamily Asteroideae), in addition to *A. lappa* and *S. taccada* used in the initial analyses. This sampling covers three divergent Asteraceae lineages and the outgroup Goodeniaceae.

For the expanded phylogenetic analyses, syntenic orthologs across the four species were retrieved from the GENESPACE results. Orthologous groups missing copies in no more than two species were used for gene tree inference. DNA sequences were aligned and cleaned as described above, and alignments longer than 300 sites were used for gene tree building using RAxML-NG v1.2.0 (*--model GTR*) (8). Coalescent trees for each set of AGBs were reconstructed using ASTRAL v5.7.8 (10), with quartet support (*-t 1*) and local posterior probability (*-t 3*) estimated independently. To assess concordance and discordance in the phylogenies, gene concordance factor (gCF) and site concordance factor (sCF) (11, 12) were calculated using the coalescent tree as the reference and the best ML trees as inputs. These calculations were performed in IQ-TREE v3.0.1 (*--scfl 100*) (13).

Synonymous substitutions analysis

Synonymous substitutions per site (*Ks*) can be used to infer the divergence of gene sequences descent from a common ancestral gene. First, syntenic gene pairs within the AGBs were used to calculate ingroup *Ks* values. Second, syntenic gene pairs between the AGBs and *S. taccada* were used to calculate ingroup-outgroup *Ks* values. The CDS sequences of syntenic gene pairs were aligned using MUSCLE v5.3 (14) in *codon* mode, and *Ks* values were calculated using the *ksd* function in Wgd v2.0.38 (15) using default settings. *Ks* calculations were conducted for each block of the 16 x 3 AGBs. *Ks* distributions were visualized using histogram and violin plots, and the significance of divergence between groups was assessed using Tukey's HSD for multiple comparisons of means.

Identification of retained triplicated genes (RTGs)

To perform the analysis, the proteomes of the species used in this study were subjected to phylogenetic profiling in OrthoFinder v2.5.5 (3) with a predefined phylogenetic topology. Hierarchical orthologous groups at node N1 (Fig. S1) were used to identify paleoparalogs through synteny analysis, as described in the microsynteny analysis section. The strictest criterion would require the presence of all three paleoparalogs in all species. However, to account for incompleteness in genome assemblies and annotations (although most genomes used here have BUSCO scores >90), two relaxed criteria were applied to identify the RTGs.

First, a gene was retained if all three paleoparalogs were present in more than 10 of the 14 species that show no evidence of subsequent WGD after the ancient WGT. Second, a gene was retained if all three paleoparalogs were present in more than 75% of species within each of the three

Asteraceae subfamilies. The intersection set from the two sets was taken as the final set of RTGs. RTGs density was calculated as the number of RTGs per 50 genes, using AGBs as a reference, and visualized as polygon plots with *RIdeogram* (16).

Frackify analyses

To further validate fractionation patterns, we ran the Frackify pipeline, which identifies paleoparalogous gene pairs from interspecific and intraspecific syntenic comparisons (17). Syntenic comparisons were performed in Asteraceae species using *Scaevola taccada* as an outgroup, with default settings for MCScanX (18). For each collinear gene pair, *Ks* values were calculated using the *add_ka_and_ks_to_collinearity.pl* script from the MCScanX repository. Peaks in the *Ks* distributions were then identified using the *find_peaks*, *nparam_density*, and *gaussian_kde* functions from the Numpy and Scipy python libraries (19, 20). These data were subsequently used as input for Frackify (17).

Gene expression, GO and KEGG enrichment analyses

RNA-seq reads were mapped to the reference genome using STAR v2.7.11b with default settings (21), and TPM (transcripts per million) values were calculated using TPMcalculator v0.0.5 (22). Genes with TPM ≥ 5 in at least one tissue were retained for further analysis. We applied this cutoff to filter out lowly expressed genes because gene expression specificity analysis is insensitive to expression level, and including lowly expressed genes could lead to misleading interpretations. Gene expression specificity (*tau* factor) was estimated used an extended *tau* score algorithm with default settings (23).

Overrepresented GO (Gene Ontology) and KEGG (Kyoto Encyclopedia of Genes and Genomes) terms in RTGs were identified using Fisher's exact test, and the significance (adjusted *p* value) was estimated using the Benjamini-Hochberg method (24) to account for multiple testing. Lettuce (*Lactuca sativa*) used as a reference for functional enrichment analyses. Protein-coding genes were first functionally annotated using eggNOGMapper (http://eggnog-mapper.embl.de/), and a custom lettuce-specific database linking gene IDs to GO and KEGG terms was built using the script: *1_build_OrgDB.R* (https://github.com/xiaoyezao/Alpine-Plant-Genomics). Overrepresented GO and KEGG terms in gene sets of interest, including conserved syntenic genes or the RTGs, were explored using clusterProfiler v4.0 (25) via the pipeline script: *2_GO_KEGG.R* (https://github.com/xiaoyezao/Alpine-Plant-Genomics). Enrichment analyses were performed using the *enricher* function (*pvalueCutoff = 0.05*, *qvalueCutoff = 1*) in clusterProfiler. Overrepresented GO and KEGG terms were visualized as bar plots, and overlapping or related terms were clustered using *the pairwise_termsim* function in clusterProfiler.

SI Tables

Table S1. The genome assemblies used in this study, and their quality assessments based on BUSCO and OMArk.

Species	Reference	OMArk	BUSCO
Arctium lappa	Fan et al. 2022 MER	97.76	98.0
Artemisia annua	Liao et al. 2022 Molecular Plant	94.91	95.5
Artemisia argyi	Chen et al. 2022 Plant Commun.	94.86	97.1
Artemisia tridentata	Melton et al. 2022 G3	73.18	89.5
Bidens hawaiensis	Bellinger et al. 2022 Journal of Heredity	80.05	96.6
Carthamus tinctorius	Wu et al. 2021 Plant Biotechnol J.	90.75	97.9
Chrysanthemum lavandulifolium	Wen et al. 2022 Horticulture Research	91.65	90.4
Chrysanthemum morifolium	Song et al. 2023 Nat Commun	97.64	98.2
Chrysanthemum nankingense	Song et al. 2018 Molecular Plant	92.68	85.8
Chrysanthemum seticuspe	Nakano et al. 2021 Commun Biol.	96.25	97.4
Cichorium endivia	Fan et al. 2022 MER	97.97	98.2
Cichorium intybus	Fan et al. 2022 MER	97.71	98.0
Conyza canadensis	Laforest et al. 2020 Pest Manag Sci	91.88	96.8
Cynara cardunculus	Acquadro et al. 2020 G3	96.31	97.6
Erigeron breviscapus	He et al. 2021 Mol Ecol Resour.	82.94	88.5
Glebionis coronaria	Wang et al. 2022 DNA Research	97.01	96.9
Helianthus annuus	Badouin et al. 2017 Nature	98.12	97.4
Lactuca saligna	Xiong et al. 2023 Plant J	94.28	92.4
Lactuca sativa	Reyes-Chin-Wo et al. 2017 Nat Commun.	94.24	98.2
Lactuca virosa	Xiong et al. 2023 G3	90.36	96.4
Mikania micrantha	Liu et al. 2020 Nat Commun	92.29	94.3
Pluchea_indica	He et al. 2022 Nat Ecol Evol	90.95	97.7
Saussurea obvallata	Zhang et al. 2023 JIPB	94.37	93.5
Scaevola_taccada	He et al. 2022 Nat Ecol Evol	93.08	95.5
Scalesia atractyloides	Cerca et al. 2022 Nat Commun	82.54	97.4
Smallanthus sonchifolius	Fan et al. 2022 MER	97.93	98.2
Stevia rebaudiana	Xu et al. 2021 Horticulture Research	96.53	96.6
Tagetes erecta	Xin et al. 2023 Horticultural Plant Journal	95.82	97.4
Taraxacum kok-saghyz	Lin et al. 2022 Sci. China Life Sci.	91.67	94.0
Taraxacum mongolicum	Lin et al. 2022 Sci. China Life Sci.	89.08	97.1
Taraxacum officinale	Xiong et al. 2023; Front. Pl. Sci.	97.48	97.6

Table S2. The number of triple-, double- and single-copy genes in selected Asteraceae species identified by Frackfy analysis.

Species	Ortholog Divergence	Non-Plaleologs	Single	Double	Triple
Arctium lappa	1.55	35316	6552	4404	783
Carthamus tinctorius	1.39	24329	5415	2764	831
Chrysanthemum lavandulifolium	1.32	50739	9227	3778	513
Chrysanthemum seticuspe	1.39	59936	13319	420	177
Cichorium endivia	1.47	49714	7378	5324	1365
Cichorium intybus	1.48	39570	7679	5338	1359
Conyza canadensis	1.36	34204	10817	420	33
Cynara cardunculus	1.39	18541	5267	3716	1107
Lactuca sativa	1.43	40063	4263	1572	2698
Lactuca virosa	1.42	30655	5825	2820	588
Taraxacum kok-saghyz	1.40	36168	5678	2778	600
Taraxacum mongolicum	1.34	38497	4637	1952	465
Glebionis coronaria	1.33	65618	6646	3232	594
Saussurea obvallata	1.47	28334	5388	3286	930

Table S3. The preliminary genomic blocks reconstructed by synteny analysis *of Arctium lappa* and *Scaevola taccada* genome.

AGB	Preliminary block	Chromosome	Start	End
a1	1a	Chr01	529296	21476215
b1	1b	Chr09	75937847	89217519
c1	1c1	Chr15	65721354	69485846
	1c2	Chr07	82143239	87239967
a2	2a1	Chr08	365772	15521861
	2a2	Chr08	16846131	16943359
	2a3	Chr08	81318833	81544418
b2	2b	Chr10	3880103	20690024
c2	2c1	Chr03	22266334	23965949
	2c2	Chr03	51382934	51701842
	2c3	Chr03	60251536	77562846
	2c4	Chr03	110448553	116707411
a3	3a1	Chr13	3409184	11749833
	3a2	Chr13	21380611	24993262
	3a3	Chr13	63533310	72922536
b3	3b1	Chr06	56019928	66687867
	3b2	Chr06	70628259	70888880
	3b3	Chr06	75554921	81102966
c3	3c1	Chr10	39892445	42035765
	3c2	Chr10	51458932	73262724
	3c3	Chr11	72273330	76174000
a4	4a	Chr07	295639	57036683
b4	4b1	Chr01	86454250	87498531
	4b2	Chr01	120430365	156434504
	4b3	Chr01	171495731	173450633
c4	4c	Chr09	43842	29978977
a5	5a	Chr02	93251042	157603240
b5	5b1	Chr15	46973148	65568283
	5b2	Chr07	87293865	90776860
c5	5c1	Chr12	34505	7174774
	5c2	Chr12	7789903	27042477
a6	6a	Chr07	60972026	81159671
b6	6b	Chr01	21710652	73925159
c6	6c	Chr09	30361316	75137089
a7	7a1	Chr16	3406657	7950589
	7a2	Chr16	16359267	20329353
	7a3	Chr16	51121676	67085749
b7	7b1	Chr03	78127521	85615464
	7b2	Chr03	100415507	110066322
	7b3	Chr03	118039180	118092500
c7	7c1	Chr18	1642163	24608345
	7c2	Chr18	49883233	60692528
a8	8a1	Chr02	5021797	5084630
	8a2	Chr02	7263569	11288955
	8a3	Chr02	54843683	82516612
b8	8b	Chr15	723740	42891037
c8	8c	Chr12	29880019	75561773

a9	9a1	Chr13	80236	3191392
	9a2	Chr13	12082949	12235910
	9a3	Chr13	25404005	56090440
b9	9b1	Chr06	39685873	52587089
	9b2	Chr06	68235247	68765717
	9b3	Chr06	71442908	73790427
	9b4	Chr06	81225927	82173711
c9	9c1	Chr10	24407866	39518440
	9c2	Chr10	48195948	49567144
	9c3	Chr10	78016215	83000109
	9c4	Chr11	68235370	70389505
a10	10a	Chr03	118490988	137430348
b10	10b	Chr04	29666688	61971366
c10	10c1	Chr02	136534	1670477
	10c2	Chr02	3248650	4785067
	10c3	Chr02	11990800	35093058
a11	11a	Chr14	771653	70387141
b11	11b	Chr17	430049	61897222
c11	11c1	Chr01	92594094	119199863
	11c2	Chr01	157650088	171233085
	11c3	Chr01	173677692	179768369
a12	12a1	Chr04	467287	21007176
	12a2	Chr04	97293500	115772556
b12	12b1	Chr06	10482926	38464549
	12b2	Chr06	83717953	97867094
c12	12c1	Chr05	16707365	71738454
	12c2	Chr05	79408044	80388029
a13	13a1	Chr05	431053	16402359
	13a2	Chr05	78716097	78836232
	13a3	Chr05	81860854	102544045
b13	13b1	Chr08	17041479	64457539
	13b2	Chr08	83796182	87820168
c13	13c1	Chr10	272485	3683738
	13c2	Chr11	434973	36484033
a14	14b1	Chr02	1701229	2200423
	14b2	Chr02	5218593	6720911
b14	14a1	Chr03	304426	17076419
c14	14c1	Chr04	22641640	27519280
	14c2	Chr04	67868485	96467356
a15	15a	Chr03	87445957	98769830
b15	15b	Chr18	27778967	48970569
c15	15c1	Chr16	186736	3101824
	15c2	Chr16	14545203	14621728
	15c3	Chr16	24589206	49174795
a16	16a1	Chr03	32192925	46498219
	16a2	Chr03	52228914	54783387
b16	16b1	Chr08	16258531	16493451
	16b2	Chr08	73052922	79740116
c16	16c1	Chr10	22168684	24181978
	16c2	Chr11	40691334	67944495

SI Figures

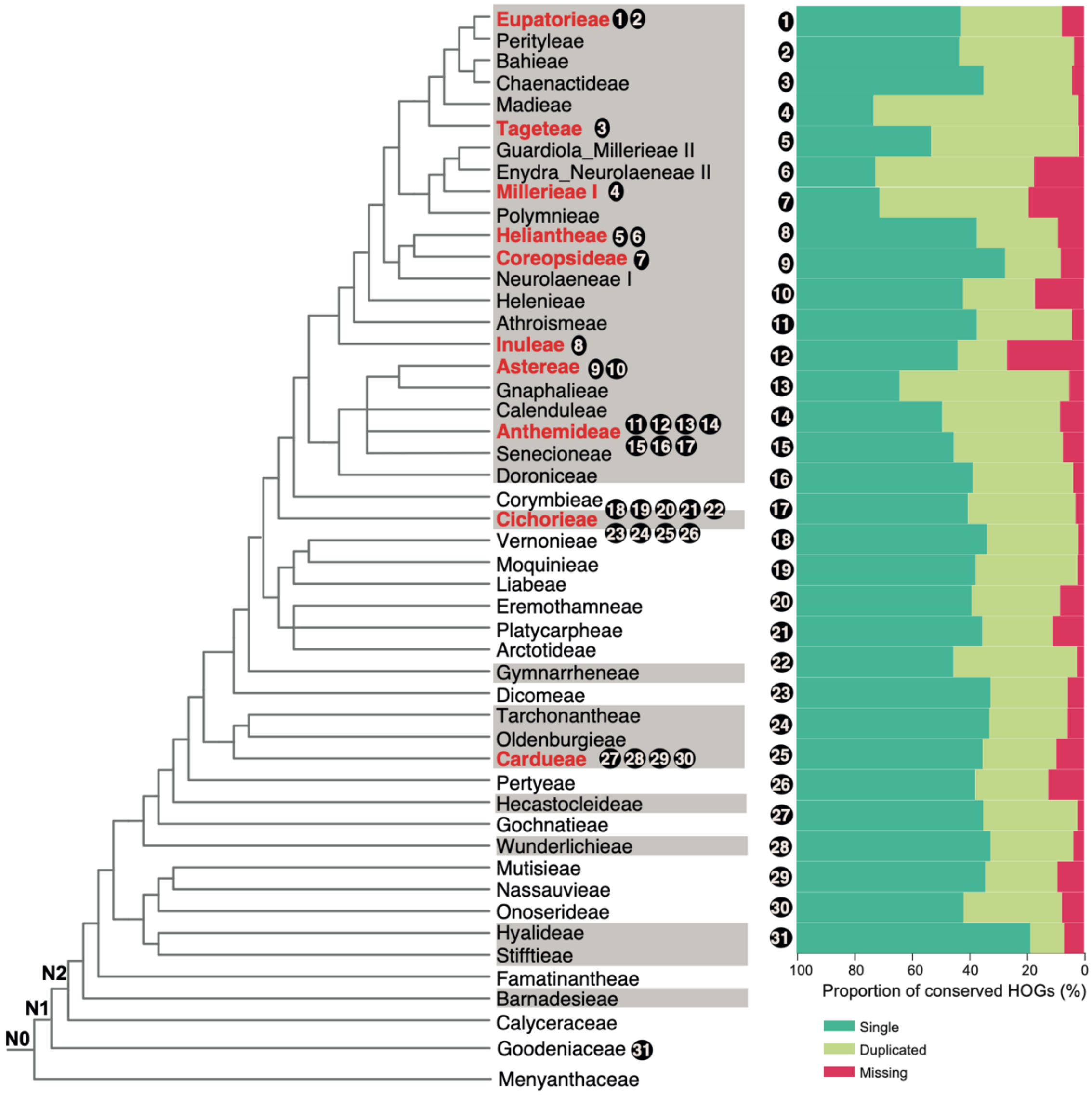

Fig. S1. Thirty genomes from 10 tribes in three subfamilies of Asteraceae, and one from the sister family Goodeniaceae are mapped to the most updated tribe-level Asteraceae phylogeny (left), with genome quality assessment results (right). 1-*Mikania micrantha*; 2-*Stevia rebaudiana*; 3-*Tagetes erecta*; 4-*Smallanthus sonchifolius*; 5-*Helianthus annuus*; 6-*Scalesia atractyloides*; 7-*Bidens hawaiensis*; 8-*Pluchea indica*; 9-*Conyza canadensis*; 10-*Erigeron breviscapus*; 11-*Artemisia annua*; 12-*Artemisia tridentata*; 13-*Artemisia argy*; 14-*Chrysanthemum lavandulifolium*; 15-*Chrysanthemum nankingense*; 16-*Chrysanthemum seticuspe*; 17-*Glebionis coronaria*; 18-*Cichorium endivia*; 19-*Cichorium intybus*; 20-*Taraxacum kok-saghyz*; 21-*Taraxacum mongolicum*; 22-*Taraxacum officinale*; 23-*Lactuca saligna*; 24-*Lactuca sativa*; 25-*Lactuca virosa*; 26-*Chrysanthemum morifolium*; 27-*Arctium lappa*; 28-*Cynara cardunculus*; 29-*Carthamus tinctorius*; 30-*Saussurea obvallata*; 31-*Scaevola taccada*.

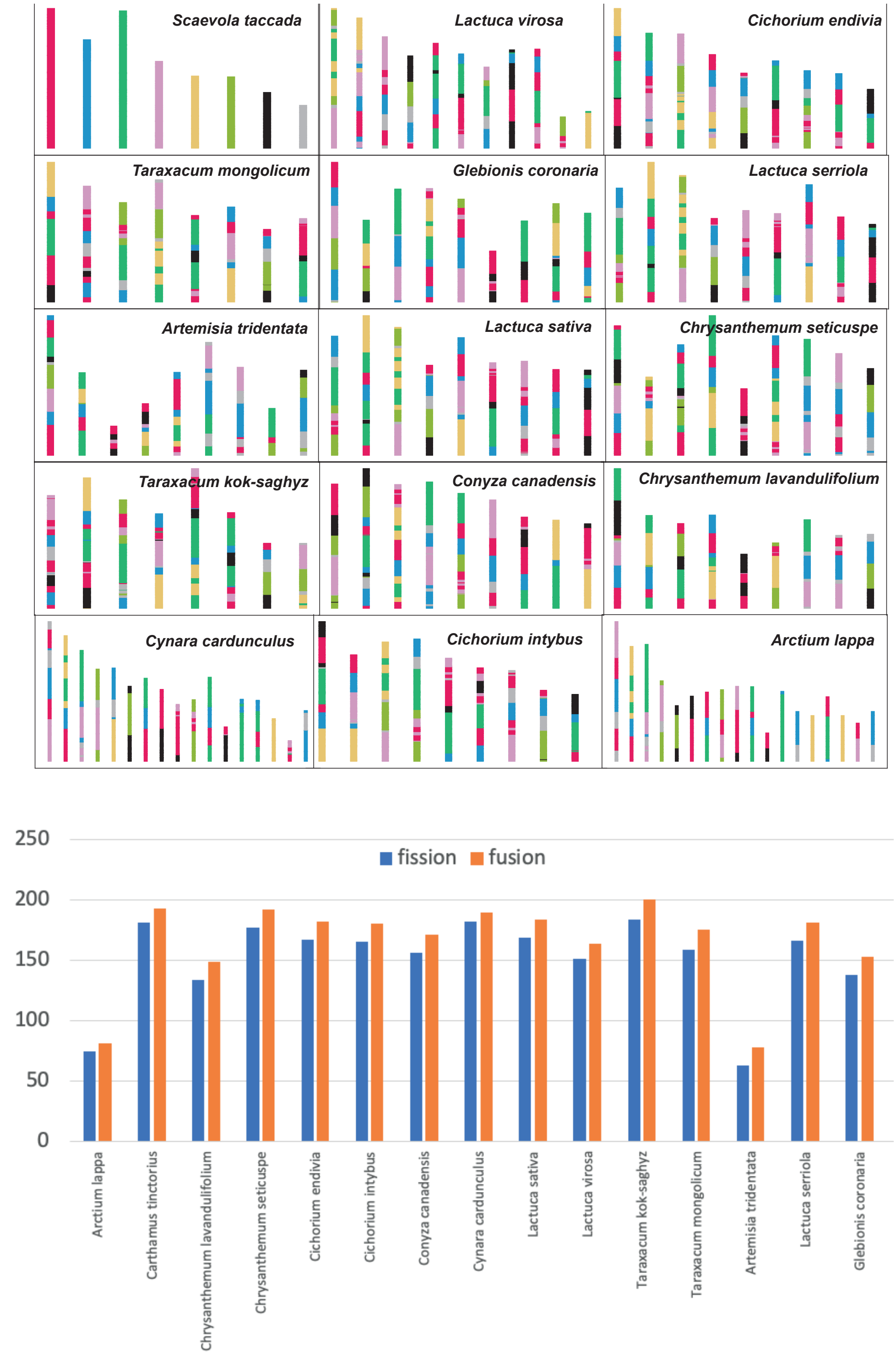

Fig. S2. Genome rearrangements from the *Scaevola taccada* genome to diploid Asteraceae genomes. **Top:** Genome architectures of *S. taccada* and representative Asteraceae species, with genomic segments colour-coded to trace the inheritance of ancestral genomic regions; **Bottom:** Quantification of chromosomal fission and fusion events from *S. taccada* to each Asteraceae species.

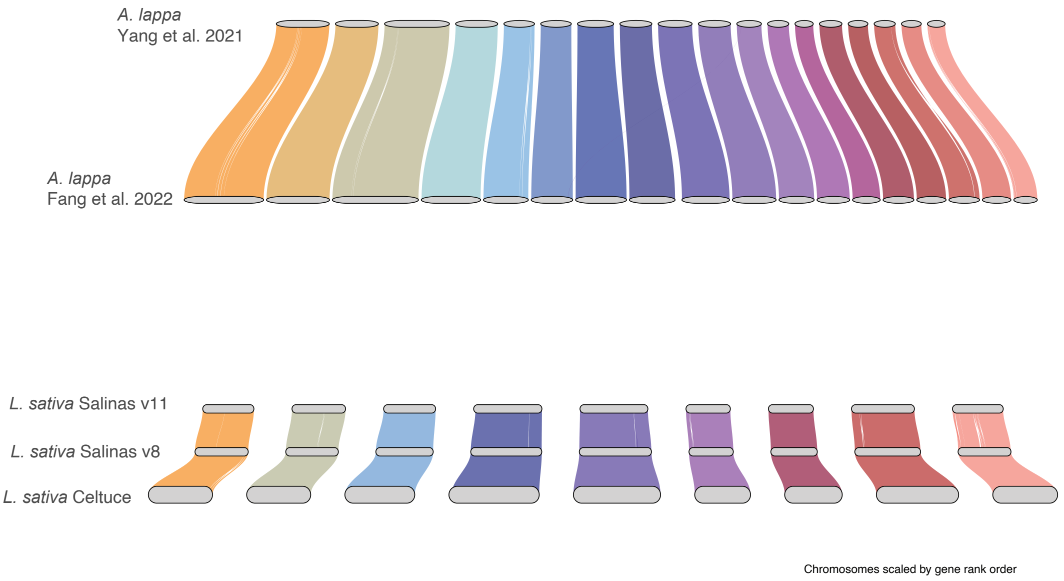

Fig. S3. Globe synteny between two *Arcticum lappa* genome assemblies Yang 2021 (26) and Fan 2022 (27) (**Top**), and three *Lactuca sativa* genome assemblies: Salinas v11, Salinas v8 and Celtuce (**Bottom**).

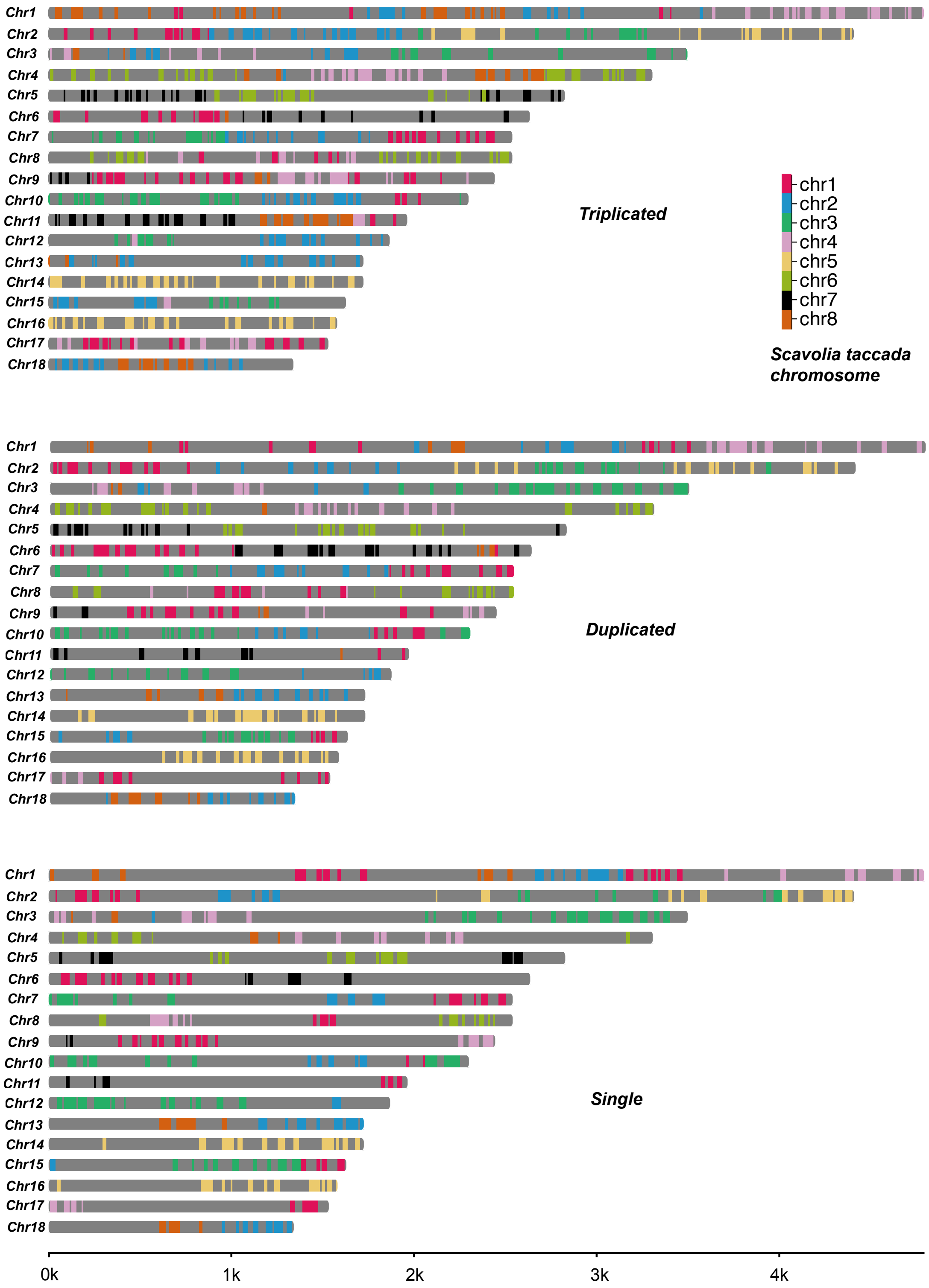

Fig. S4. Genomic architecture of *Arctium lappa* (18 chromosomes) illustrated in the context of synteny blocks identified through comparison with the *Scaevola taccada* genome. Synteny blocks are categorized into triplicated (top), duplicated (middle) and single copy (bottom), according to the copy number ratio of homologous blocks between *A. lappa* and *S. taccada*. Block colours represent their corresponding homologous positions on *S. taccada* chromosomes.

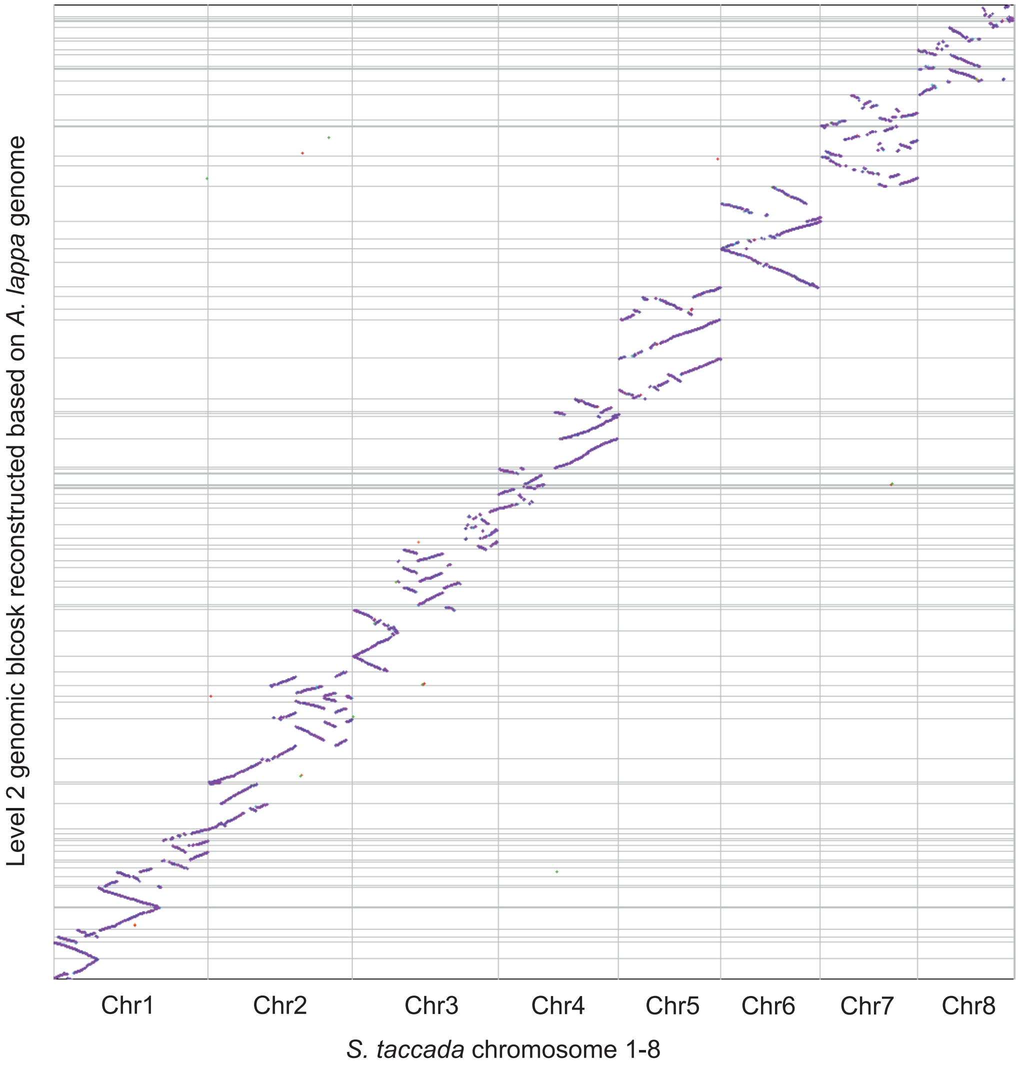

Fig. S5. Genomic dot plot of the level 2 genomic blocks and *Scaevola taccada* genome. The X-axis represents the eight chromosomes of the *S. taccada* genome, and Y-axis shows the level 2 blocks reconstructed from the *Arctium lappa* genome.

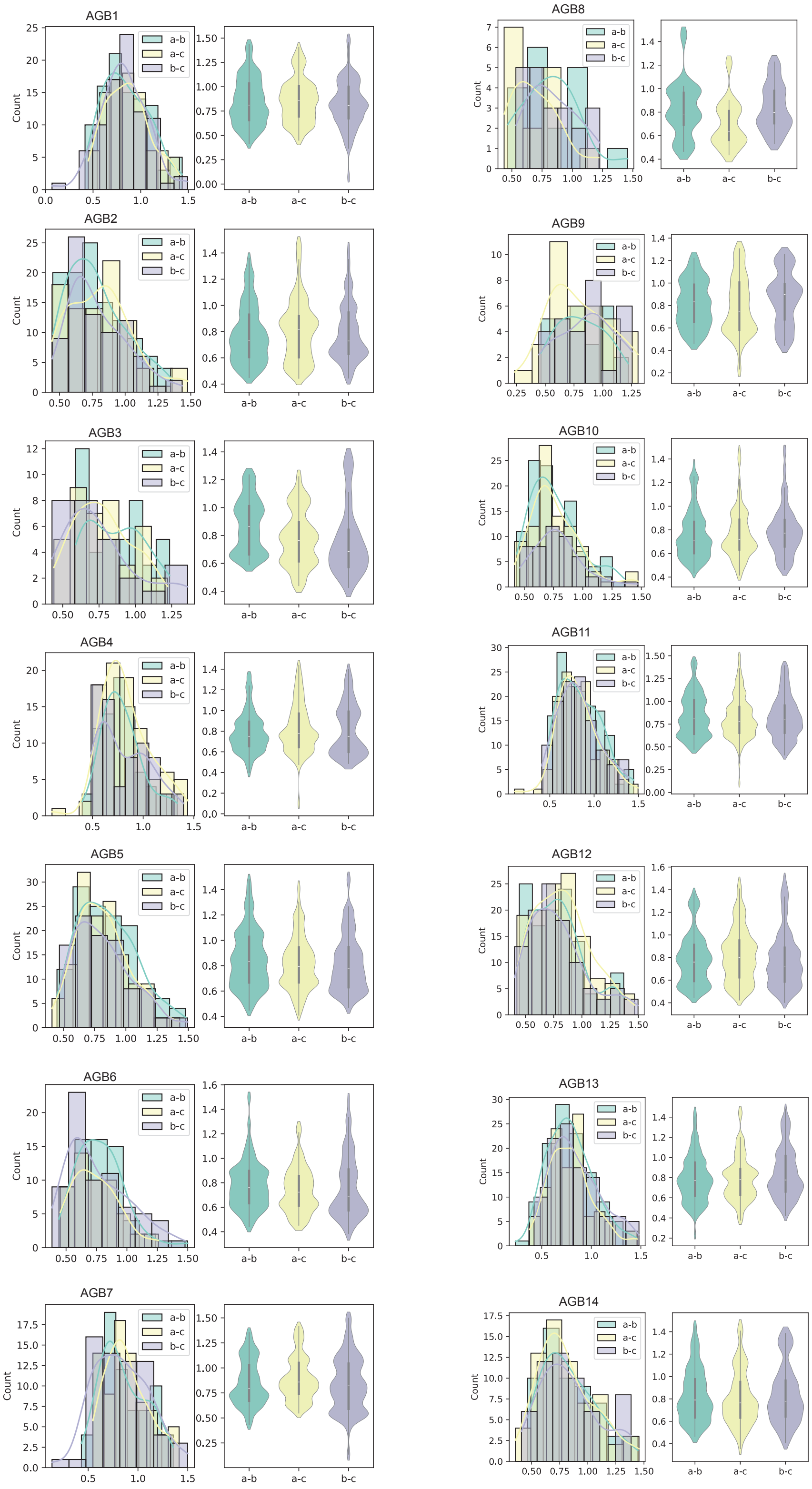

Fig. S6. Distribution of synonymous substitution rates (*Ks*) among syntenic gene pairs between homoeologous blocks within AGB1-16.

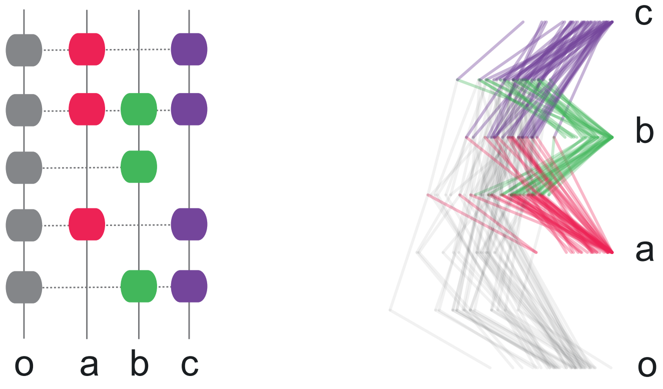

Fig. S7. Phylogenomic analysis of subgenomes. **Left**: Diagram illustrating a window of syntenic genes from the subgenome *a*, *b*, *c* and outgroup *o*; **Right**: DensiTree visualization of phylogenies inferred from syntenic genes in AGB11.

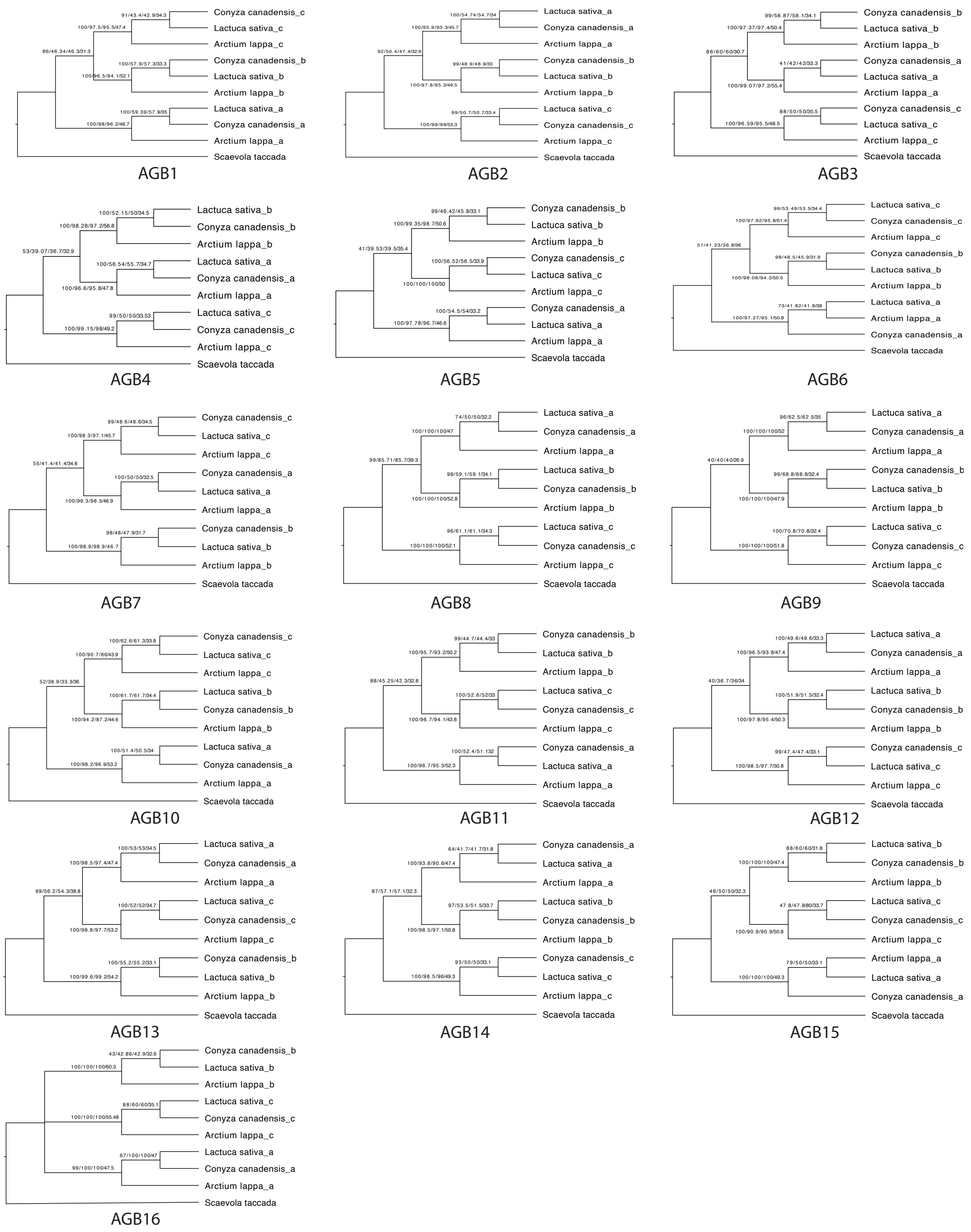

Fig. S8. Coalescent phylogeny of subgenomes with AGBs 1-16, reconstructed from maximum likelihood (ML) trees of syntenic genes within each AGBs. Node labels indicate local posterior probability, quartet support, gene concordance factor (gCF), and site concordance factor (sCF). Stem branch lengths are shown in ASTRAL coalescent units. The inferred position of the genome triplication event is highlighted with a grey bar.

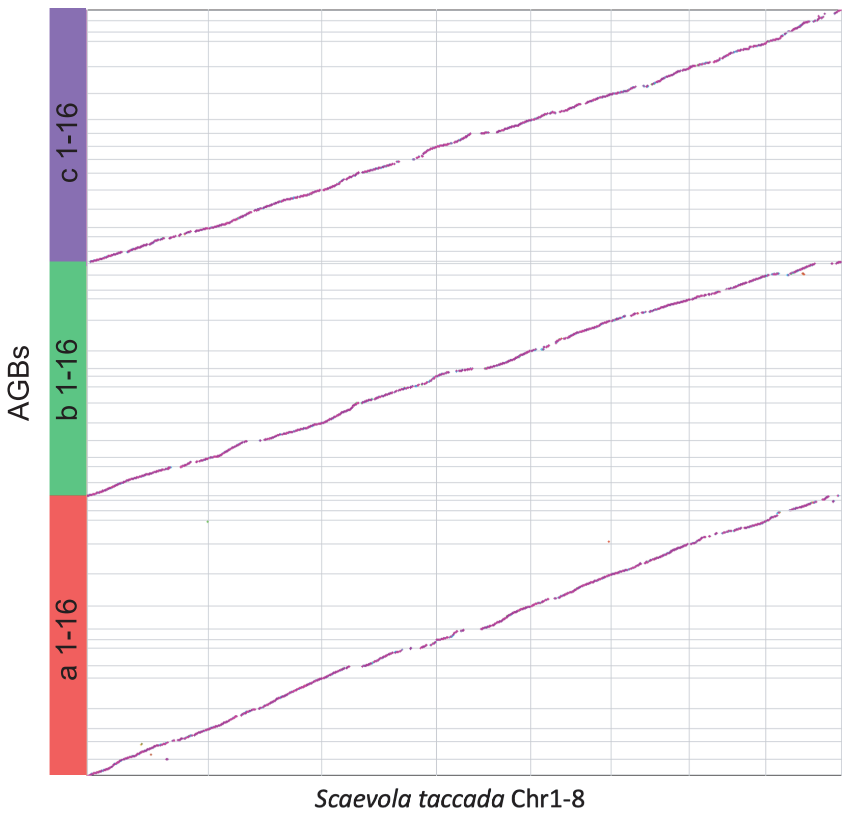

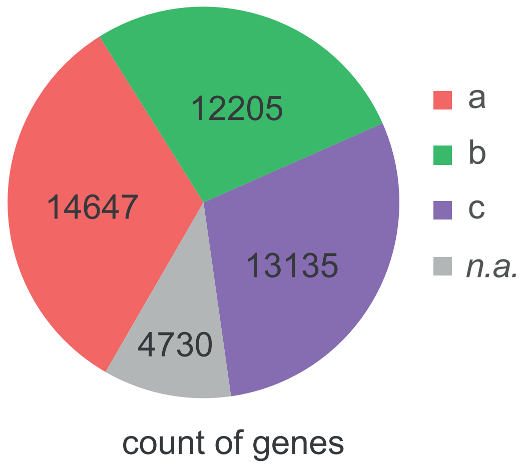

Fig. S9. Asteraceae genomic blocks (AGBs) reconstructed from preliminary genomic blocks identified in the *Arctium lappa* genome. **Top:** Genomic dot plot between *Scaevola taccada* (X-axis) and the AGBs (Y-axis). **Bottom:** Gene counts in the a, b and c subcategories of the AGBs. "*n.a.*" indicates genes present in the *Arctium lappa* genome but absent from the AGBs.

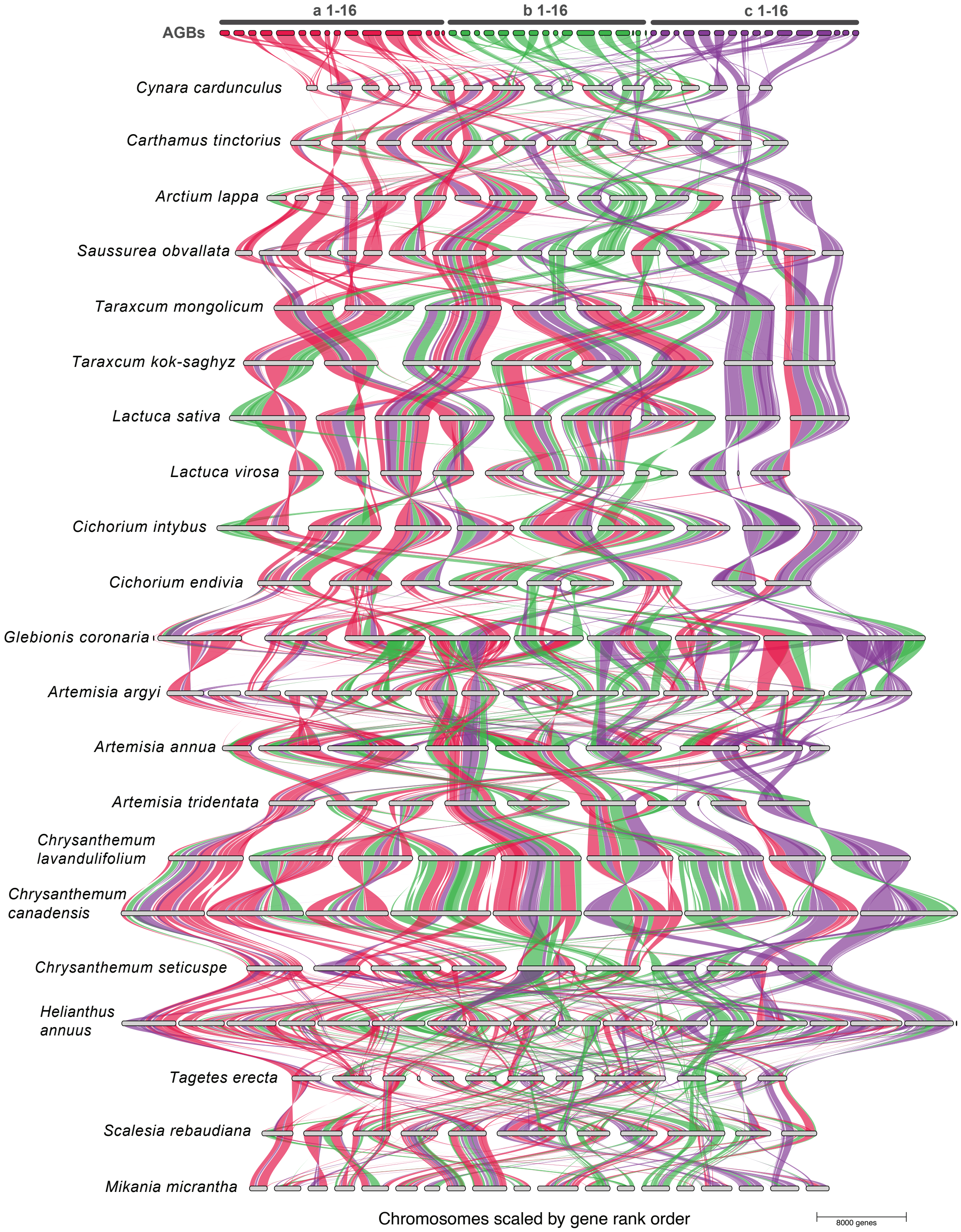

Fig. S10. Macrosynteny (riparian plot) across Asteraceae genomes in the context of AGBs. The AGBs are shown at the top and highlighted in red, green and purple to visualize their conservation and rearrangements across different Asteraceae species.

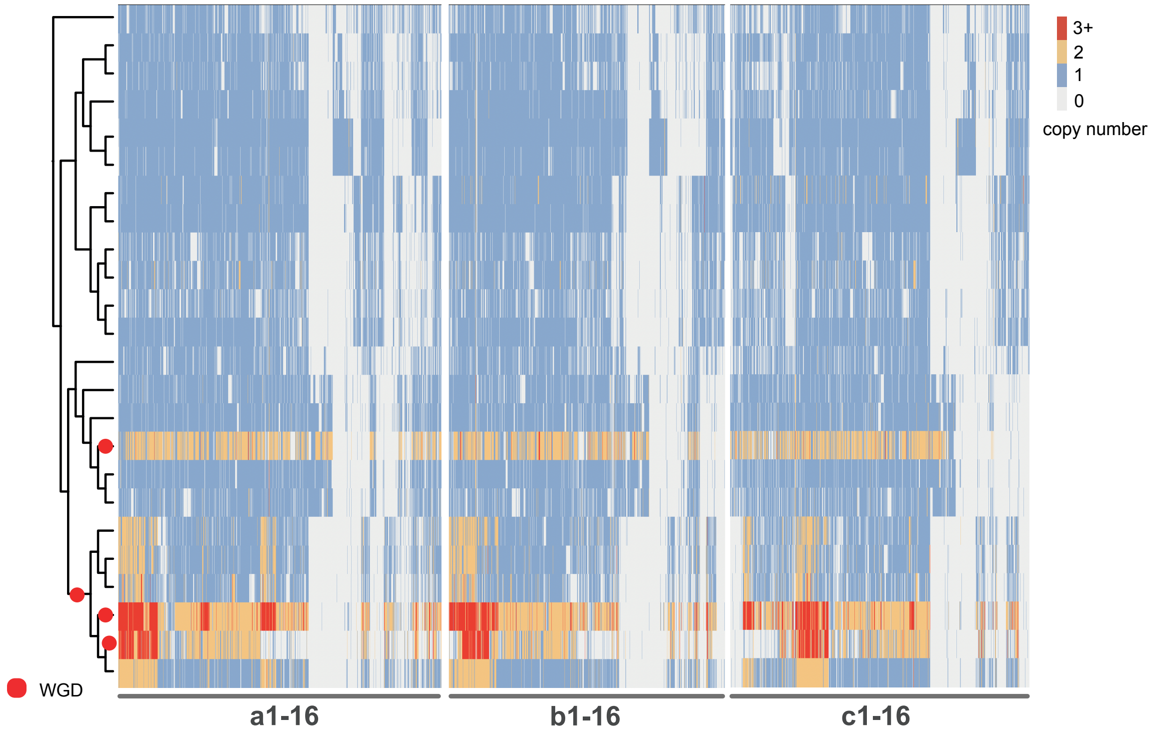

Fig. S11. Microsynteny across Asteraceae genomes in context of AGBs. Rows represent species (in the same order as in Fig. S9), and columns represent synteny clusters, each comprising homologous genes that are syntenic across two or more species. Gene copy number is indicated by colour bars. Presence/absence patterns are clustered based on Euclidean distance.

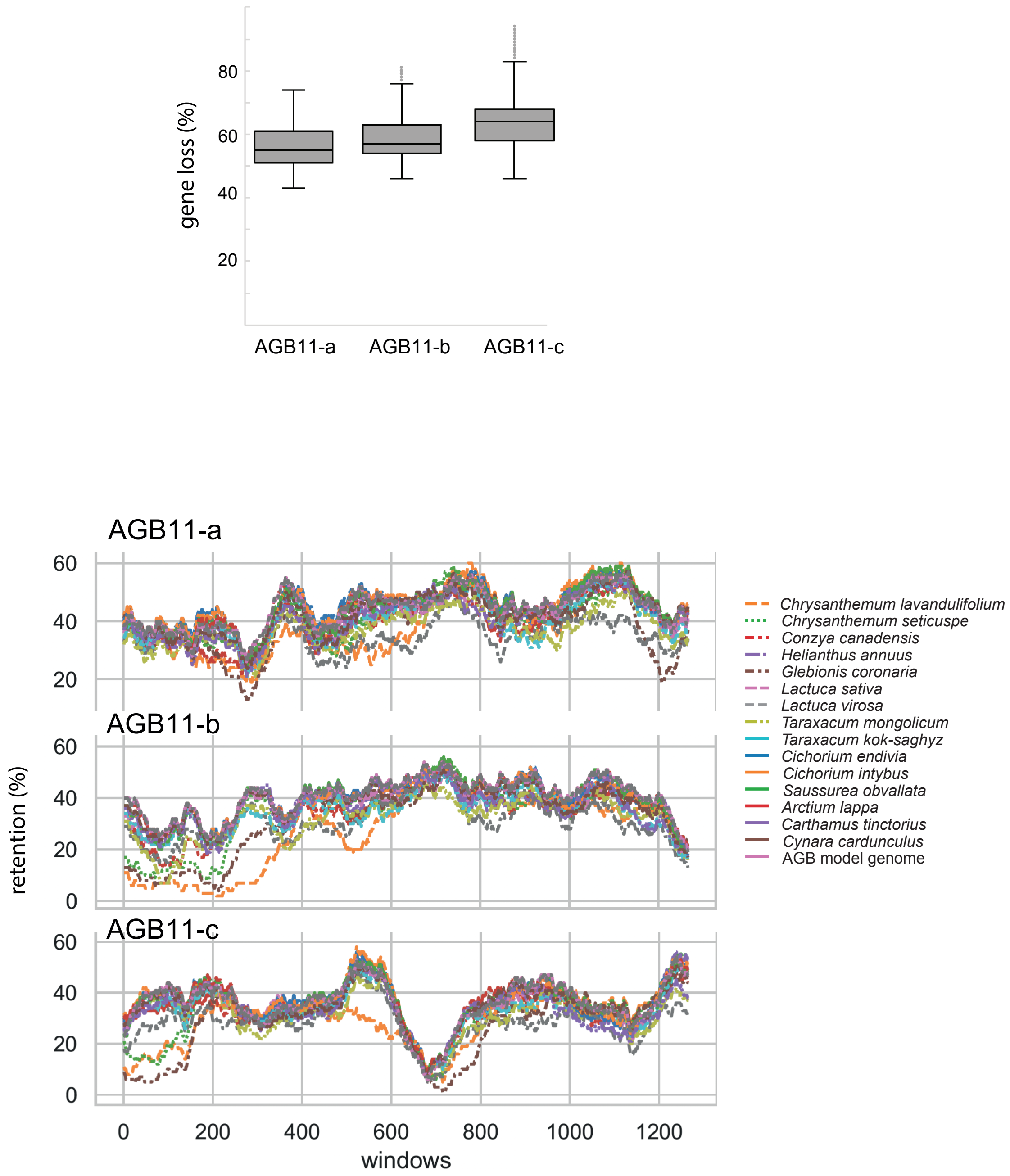

Fig. S12. Gene fractionation patterns on AGB11 in selected Asteraceae species. **Top:** Box plot showing gene loss rates on the three homoeologous blocks. **Bottom:** Gene fractionation patterns, represented by gene retention rates of syntenic genes calculated in 100-gene windows, using *Scaevola taccada* as the reference.

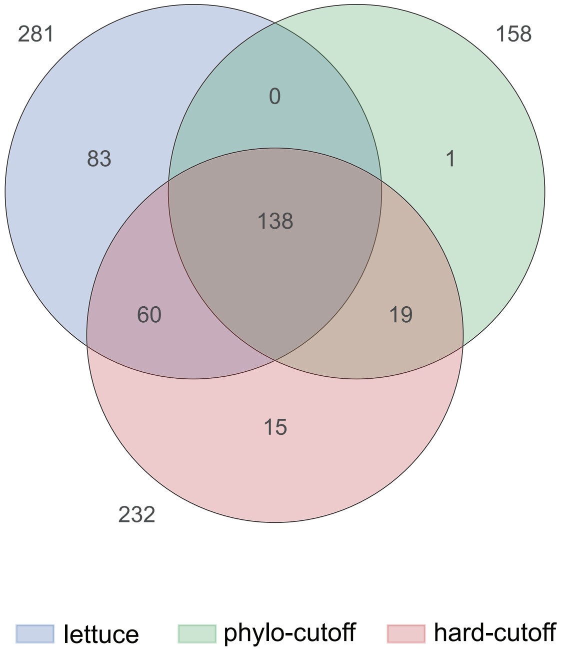

Fig. S13. Retained triplicated genes under different cutoffs. *Lettuce:* Genes with all three copies retained in *Lactuca sativa*; *Phylo-cutoff*: Genes present in more than 75% of species within each of the three Asteraceae subfamilies; *Hard-cutoff*: Genes present in more than 10 out of 14 species in total.

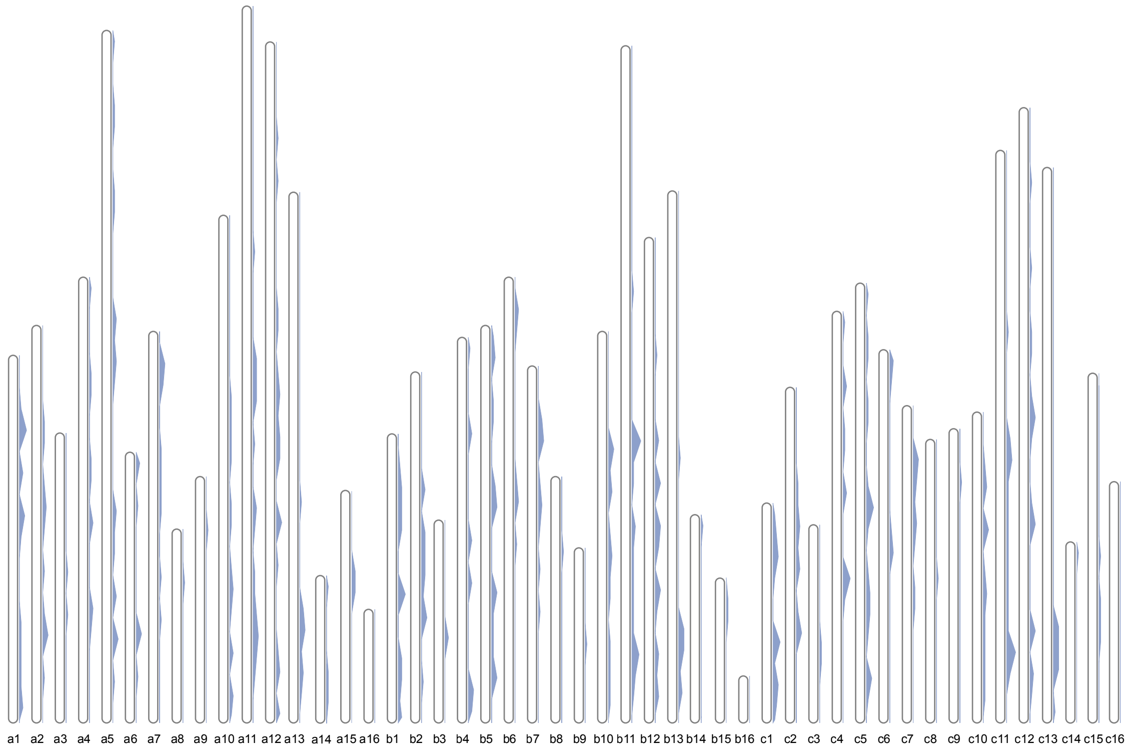

Fig. S14. Density of retained triplicated genes (RTGs) across the 16 x 3 Asteraceae genomic blocks (AGBs).

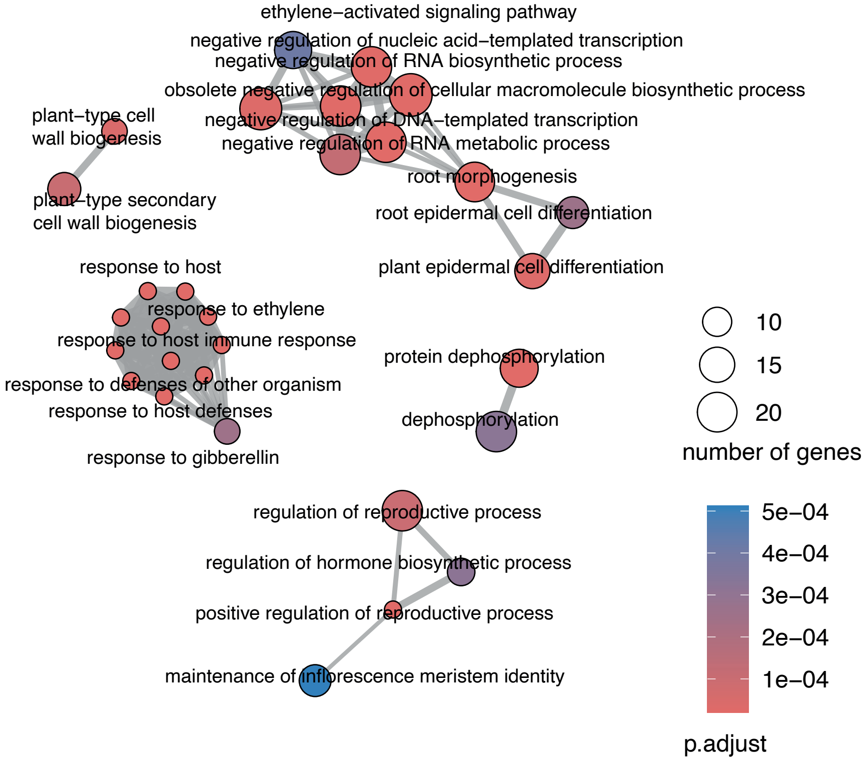

Fig. S15. Significantly enriched Gene Ontology (GO) terms among the 157 retained triplicated genes (RTGs).

SI Datasets (separate files)

Dataset S1. Gene fractionation across Asteraceae genomic blocks (AGBs). Fractionation rates were calculated based on syntenic gene pairs within 100-gene windows, using the *Scaevola taccada* genome as the reference.

Dataset S2. The retained triplicated genes (RTGs) identified by phylogenomic synteny analysis.

SI References

1. M. Seppey, M. Manni, E. M. Zdobnov, "BUSCO: Assessing genome assembly and annotation completeness" in *Methods in Molecular Biology*, (2019), pp. 227–245.
2. Y. Nevers, *et al.*, Quality assessment of gene repertoire annotations with OMArk. *Nat Biotechnol* 1–17 (2024). https://doi.org/10.1038/s41587-024-02147-w.
3. D. M. Emms, S. Kelly, OrthoFinder: Phylogenetic orthology inference for comparative genomics. *Genome Biol* **20**, 1–14 (2019).
4. J. T. Lovell, *et al.*, GENESPACE tracks regions of interest and gene copy number variation across multiple genomes. *Elife* **11**, 1–20 (2022).
5. K. Katoh, D. M. Standley, MAFFT multiple sequence alignment software version 7: Improvements in performance and usability. *Mol Biol Evol* **30**, 772–780 (2013).
6. S. Capella-Gutiérrez, J. M. Silla-Martínez, T. Gabaldón, trimAl: A tool for automated alignment trimming in large-scale phylogenetic analyses. *Bioinformatics* **25**, 1972–1973 (2009).
7. S. Kalyaanamoorthy, B. Q. Minh, T. K. F. Wong, A. von Haeseler, L. S. Jermiin, ModelFinder: fast model selection for accurate phylogenetic estimates. *Nat Methods* **14**, 587–589 (2017).
8. A. M. Kozlov, D. Darriba, T. Flouri, B. Morel, A. Stamatakis, RAxML-NG: a fast, scalable and user-friendly tool for maximum likelihood phylogenetic inference. *Bioinformatics* **35**, 4453–4455 (2019).
9. R. R. Bouckaert, DensiTree: making sense of sets of phylogenetic trees. *Bioinformatics* **26**, 1372–1373 (2010).
10. C. Zhang, S. Mirarab, ASTRAL-Pro 2: ultrafast species tree reconstruction from multi-copy gene family trees. *Bioinformatics* **38**, 4949–4950 (2022).
11. B. Q. Minh, M. W. Hahn, R. Lanfear, New Methods to Calculate Concordance Factors for Phylogenomic Datasets. *Mol Biol Evol* **37**, 2727–2733 (2020).
12. Y. K. Mo, R. Lanfear, M. W. Hahn, B. Q. Minh, Updated site concordance factors minimize effects of homoplasy and taxon sampling. *Bioinformatics* **39** (2023).
13. T. Wong, *et al.*, IQ-TREE 3: Phylogenomic Inference Software using Complex Evolutionary Models. [Preprint] (2025).
14. R. C. Edgar, Muscle5: High-accuracy alignment ensembles enable unbiased assessments of sequence homology and phylogeny. *Nat Commun* **13**, 6968 (2022).
15. H. Chen, A. Zwaenepoel, Y. Van de Peer, wgd v2: a suite of tools to uncover and date ancient polyploidy and whole-genome duplication. *Bioinformatics* **40** (2024).
16. Z. Hao, *et al.*, *RIdeogram* : drawing SVG graphics to visualize and map genome-wide data on the idiograms. *PeerJ Comput Sci* **6**, e251 (2020).
17. M. T. W. McKibben, M. S. Barker, "Applying Machine Learning to Classify the Origins of Gene Duplications" in *Methods in Molecular Biology*, (2023), pp. 91–119.
18. Y. Wang, *et al.*, MCScanX: a toolkit for detection and evolutionary analysis of gene synteny and collinearity. *Nucleic Acids Res* **40**, e49–e49 (2012).
19. C. R. Harris, *et al.*, Array programming with NumPy. *Nature* **585**, 357–362 (2020).
20. P. Virtanen, *et al.*, SciPy 1.0: fundamental algorithms for scientific computing in Python. *Nat Methods* **17**, 261–272 (2020).
21. A. Dobin, *et al.*, STAR: Ultrafast universal RNA-seq aligner. *Bioinformatics* **29**, 15–21 (2013).

22. R. V. Alvarez, L. S. Pongor, L. Mariño-Ramírez, D. Landsman, TPMCalculator: One-step software to quantify mRNA abundance of genomic features. *Bioinformatics* **35**, 1960–1962 (2019).
23. H. B. Lüleci, A. Yılmaz, Robust and rigorous identification of tissue-specific genes by statistically extending tau score. *BioData Min* **15**, 31 (2022).
24. Y. Benjamini, Y. Hochberg, On the adaptive control of the false discovery rate in multiple testing with independent statistics. *Journal of Educational and Behavioral Statistics* **25**, 60–83 (2000).
25. T. Wu, *et al.*, clusterProfiler 4.0: A universal enrichment tool for interpreting omics data. *The Innovation* **2**, 100141 (2021).
26. Y. Yang, *et al.*, The first high-quality chromosomal genome assembly of a medicinal and edible plant *Arctium lappa*. *Mol Ecol Resour* **22**, 1493–1507 (2022).
27. W. Fan, *et al.*, The genomes of chicory, endive, great burdock and yacon provide insights into Asteraceae palaeo-polyploidization history and plant inulin production. *Mol Ecol Resour* **22**, 3124–3140 (2022).
